# Recent Progress and Challenges of Implantable Biodegradable Biosensors

**DOI:** 10.3390/mi15040475

**Published:** 2024-03-30

**Authors:** Fahmida Alam, Md Ashfaq Ahmed, Ahmed Hasnain Jalal, Ishrak Siddiquee, Rabeya Zinnat Adury, G M Mehedi Hossain, Nezih Pala

**Affiliations:** 1 Department of Electrical and Computer Engineering, University of Texas Rio Grande Valley, Edinburg, TX 78539, USA; ahmed.jalal@utrgv.edu (A.H.J.); gmmehedi.hossain01@utrgv.edu (G.M.M.H.); 2 Baptist Health South Florida, Miami, FL 33176, USA; mdashfaq.ahmed@baptisthealth.net; 3 Institute of Microsystems Technology, University of South-Eastern Norway, Horten, 3184 Vestfold, Norway; ishrak.sdcom@gmail.com; 4 Department of Applied Physiology and Kinesiology, College of Health and Human Performance, University of Florida, Gainesville, FL 32611, USA; radury@ufl.edu; 5 Department of Electrical and Computer Engineering, Florida International University, Miami, FL 33174, USA; npala@fiu.edu

**Keywords:** implantable, biodegradable, biosensor, biocompatibility, microfabrication

## Abstract

Implantable biosensors have evolved to the cutting-edge technology of personalized health care and provide promise for future directions in precision medicine. This is the reason why these devices stand to revolutionize our approach to health and disease management and offer insights into our bodily functions in ways that have never been possible before. This review article tries to delve into the important developments, new materials, and multifarious applications of these biosensors, along with a frank discussion on the challenges that the devices will face in their clinical deployment. In addition, techniques that have been employed for the improvement of the sensitivity and specificity of the biosensors alike are focused on in this article, like new biomarkers and advanced computational and data communicational models. A significant challenge of miniaturized in situ implants is that they need to be removed after serving their purpose. Surgical expulsion provokes discomfort to patients, potentially leading to post-operative complications. Therefore, the biodegradability of implants is an alternative method for removal through natural biological processes. This includes biocompatible materials to develop sensors that remain in the body over longer periods with a much-reduced immune response and better device longevity. However, the biodegradability of implantable sensors is still in its infancy compared to conventional non-biodegradable ones. Sensor design, morphology, fabrication, power, electronics, and data transmission all play a pivotal role in developing medically approved implantable biodegradable biosensors. Advanced material science and nanotechnology extended the capacity of different research groups to implement novel courses of action to design implantable and biodegradable sensor components. But the actualization of such potential for the transformative nature of the health sector, in the first place, will have to surmount the challenges related to biofouling, managing power, guaranteeing data security, and meeting today’s rules and regulations. Solving these problems will, therefore, not only enhance the performance and reliability of implantable biodegradable biosensors but also facilitate the translation of laboratory development into clinics, serving patients worldwide in their better disease management and personalized therapeutic interventions.

## 1. Introduction

Implantable sensors are a family of medical devices that offer an efficacious pathway to real-time monitoring and therapeutic activities. Their minimally invasive implantation nature makes them ideal for in situ applications. Examples of such devices include glucose sensors for continuous monitoring in diabetic patients, pressure sensors for intracranial pressure monitoring in individuals with brain injuries, and cardiac monitors for detecting arrhythmias or monitoring heart conditions. Glucose sensors, for instance, have revolutionized diabetes management by providing patients with real-time insights into their glucose levels, enabling more precise glycemic control [1]. Similarly, implantable pressure sensors have made significant strides in neurology, aiding in the management of conditions such as hydrocephalus and traumatic brain injury by monitoring intracranial pressure [2]. Cardiac monitors, on the other hand, have been instrumental in advancing cardiology by offering continuous heart rhythm monitoring, thereby facilitating the early detection and treatment of potentially life-threatening arrhythmias [3]. These examples underscore the versatility and transformative potential of implantable sensors in addressing a wide spectrum of health conditions, heralding a new era in personalized and proactive healthcare.

Implantable sensors operate on a variety of working principles, each selected for its ability to best capture the specific physiological parameter or biomarker of interest. The most common principles are electrical, optical, chemical, and mechanical sensing mechanisms, each serving distinct purposes in medical diagnostics and monitoring. Electrical sensors are widely used for monitoring the electrical activity in tissues, such as the heart’s rhythm or brain’s electrical signals. For example, implantable cardioverter-defibrillators (ICDs) and pacemakers rely on electrical sensors to detect arrhythmias and administer appropriate electrical therapy [4,5]. Similarly, deep brain stimulation devices use electrical sensors to treat neurological disorders like Parkinson’s disease by modulating electrical signals in the brain [6]. Optical sensors employ light to measure changes in tissue properties or to detect specific biomolecules. These sensors are often used for measuring oxygen saturation in tissues, an essential parameter in critical care settings. For instance, photoplethysmography (PPG) sensors, which measure changes in blood volume using light, are used for continuous monitoring of oxygen levels [7]. Chemical sensors detect the presence or concentration of various chemical substances, including ions, gases, or biomolecules, making them invaluable for monitoring metabolic states. Glucose sensors, which measure blood sugar levels in diabetic patients, are a prime example. These sensors typically employ enzymatic reactions that generate an electrical signal proportional to the glucose concentration [1]. Mechanical sensors are used to measure physical changes, such as pressure or strain, within the body. These sensors are crucial for monitoring conditions like intracranial pressure in patients with head injuries or intraocular pressure in glaucoma management. An example includes the use of MEMS technology for creating pressure sensors that can be implanted to continuously monitor such pressures [8,9]. Each of these sensor types plays a critical role in the evolving landscape of medical diagnostics and therapeutic interventions, enabling more precise and personalized healthcare. 

However, technically challenging disposal methods of implantable sensors after usage are associated with significant complications as surgical intervention may have catastrophic outcomes. Biodegradability ensures that the implants degrade themselves naturally through biological processes while providing all the benefits of conventional implantable sensors. Their self-degradation characteristics bypass the need for surgical removal practices to alleviate patient discomfort and tissue damage. Therefore, biodegradability has become a highly anticipated subdomain of implantable biosensors and has a monumental research impact.

The purpose of biodegradable sensors is to be implanted into the human body partially or fully for a particular life cycle and to monitor relevant biomarkers, conditions, and processes without needing patient intervention. Steady monitoring of critical indications regardless of the patient’s physiological state (rest, sleep, and exercise) unravels the issue with conventional hospitalization and supervision of patients. Implantable, biodegradable sensors potentially reveal metabolic imbalances, notably diabetes, cancer, heart diseases, respiratory diseases, stroke, obesity, and numerous biomarker-based diagnoses. They are more instantaneous and cost-effective diagnosis alternatives for continuously monitoring metabolites with a minimally invasive approach. The first pacemaker was implanted by an engineer named Arne Larsson in 1958. Swedish surgeon Ake Senning and physician–inventor Rune Elmqvist were the minds behind it [10]. Since its inception, implantable sensors have been commercially available. However, most research on the biodegradability of implantable sensors has developed in the recent decade. In 2010, Irimia-Vladu et al. were among the first to publish a review dealing with biodegradable materials for organic electronics [11]. In 2021, Yeon Sik Choi et al. reported that they developed the first-ever transient pacemaker—a wireless, battery-free, fully implantable pacing device that disappears over five to seven weeks without surgical extraction [12]. Inspired by the effectiveness of minimally invasive surgery, laparoscopy, and catheter-based treatment have been adopted as standard procedures for biodegradable sensor implantation [13]. 

Materials and design choices are crucial for in situ and in vivo biological implantable devices. Functional implantable devices require the fulfillment of certain essential characteristics: (a) the devices must be environmentally friendly, biocompatible, and ideally biodegradable; (b) the substrate must be compliant and flexible with the target tissue; (c) minimum elastic difference between the device and the tissue; (d) the devices must be capable of minimally invasive implantation; and (e) the materials used for the device must be approved for human use by the Federal Drug Administration (FDA).

This article delves into the complex interplay between biocompatible materials that must coexist harmlessly within the body and the sophisticated biodegradation mechanisms that allow these devices to either dissolve or be safely removed after fulfilling their purpose. First, the article presents a section on **Materials**, that forms the cornerstone of implantable biosensor technology. Here, readers can expect a thorough analysis of biocompatible materials that are at the forefront of current research. This section aims to elucidate the properties that make materials suitable for integration into the human body without eliciting adverse reactions, highlighting the latest innovations and the ongoing quest for improved biocompatibility.

Moving forward, the article delves into the **Biodegradation Mechanisms** of these biosensors. This section promises to unpack the complex process that allows the sensor to decompose naturally within the body’s environment, mitigating the need for surgical removal and reducing potential long-term side effects. The focus here is on the delicate balance between durability for sustained operation and the capacity for safe biodegradation post-use. The intricate **Sensing Mechanisms of Biodegradable Biosensors** are explored next. Readers will gain insight into the state-of-the-art technologies that enable the conversion of biological signals into electrical ones for monitoring and diagnostics, discussing the technical nuances that ensure accuracy and reliability in the dynamic in vivo environment. A pivotal section on the **Applications of Implantable Biosensors** follows, providing a panorama of the current and potential use of these devices. From glucose monitoring to early disease detection, this part of the article showcases real-world instances where implantable biodegradable biosensors are making a significant impact, along with an exploration of emerging applications that are on the horizon. Addressing the roadblocks head-on, the **Challenges and Future Directions** section offers a critical perspective on the hurdles facing the field and anticipates where the field is headed. This includes issues such as power supply, data communication, materials, fabrication, body implantation, and long-term performance and calibration. This comprehensive overview aims to present the challenges as opportunities for innovation and growth within the sector. Looking to the horizon, we also explored the next generation of implantable biosensors, the evolution of current technologies, and the multidisciplinary research efforts that are paving the way for discoveries and applications. The article culminates with a **Conclusion** that ties together the findings and forecasts discussed, providing a succinct summary and a thoughtful reflection on the implications for the future of healthcare and biomedical technology. Through this approach, we aim to convey the complexity and dynamism of this field, equipping readers with a nuanced understanding of both its achievement and its as yet unrealized potential. 

## 2. Materials

The absence of serviceable materials limits the realization of high-performance devices for implantable biodegradable biosensors. The components, manufacturing methods, and device form factor used to make traditional biosensors are incompatible with implantable biodegradable ones. Standard biosensors, for example, are heavy and bulk in size, making them unsuitable for implantable applications that need small and light sensors for seamless body integration. Conventional sensors designed for rigid surfaces are not suitable for soft, curvilinear human tissues. Poor mechanical resilience due to the rigidity of conventional sensors may cause repeated muti-axis complex deformation which is frequently experienced by the human body [14,15].

Recognizing the inflexibility of current biosensors, direct sensing through biological tissues needs soft and stretchable sensor materials capable of conforming to the human anatomy’s nonplanar function. The implantable biodegradable biosensors must also have identical mechanical properties to the tissues so that they can be physically compatible with the contours without triggering any somatosensory response. In general, implantable biosensors are surgically removed after completing their function. This surgery may result in organ/tissue damage, or device components may be left behind in the body, which might cause post-operative complications. Hence, the solution is the selection of biodegradable materials for the implantable biosensors [16].

Recent advances have underscored the potential of polymers like poly (glycerol sebacate) (PGS) for their excellent biocompatibility and biodegradability [17], and poly(lactic-co-glycolic acid) (PLGA), known for its use in drug delivery systems and its mechanical properties akin to soft tissues [18]. Moreover, materials such as polycaprolactone (PCL) have been highlighted for their flexibility and degradation properties [19]. Textile-based sensors, incorporating conductive yarns, offer a novel approach to achieving biomechanical compatibility with skin, indicating a significant reduction in irritation and device failure [20]. Innovations in material science, such as the development of silk fibroin-based materials, present promising avenues for implantable electronics, offering strength, flexibility, and biodegradability [21]. Furthermore, research into nanocomposite materials, combining biodegradable polymers with nanoparticles, aims to enhance sensor functionality while ensuring biocompatibility and mechanical integrity [22].

**Figure 1:**
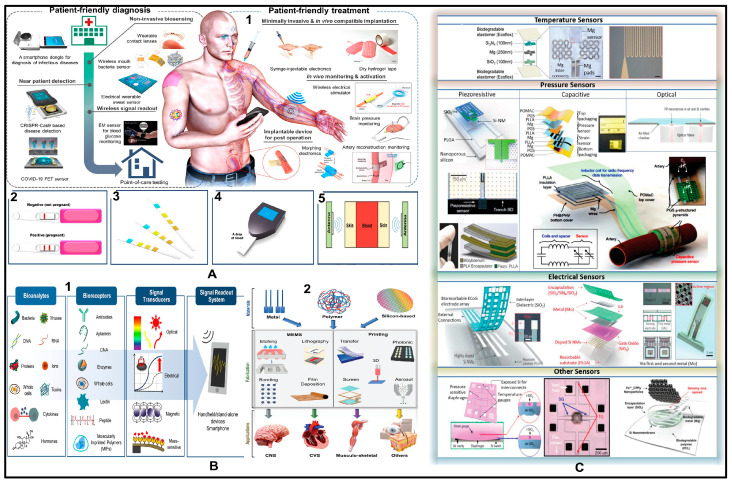
(**A**) (1) Patient-friendly diagnosis and treatment with implantable biosensors. Point of care diagnosis utilizing commercialized portable biosensors. (2) Conceptual illustration of the colorimetric pregnancy test. (3) Conceptual illustration of urine test strips (dipstick tests). (4) Conceptual illustration of the personal blood glucose meter. (5) Design concept of a millimeter (mm) wave glucose sensor. The skin/blood/skin stacked structure is placed in between the two antennas. (**B**) (1) Schematic illustration of the four basic units of biosensors. Adapted in part with permission from ref [24] under the terms of the Creative Commons Attribution CC by 4.0 (copyright 2021 American Chemical Society). (2) Overview of developments in biodegradable implantable sensors, their comprising biomaterials (metals, polymers, silicon-based semiconducting materials), fabrication techniques, and reporting applications. CNS: central nervous system; CVS: cardiovascular system silicon based. (**C**) Different methodologies to develop biosensors. Image adapted from [25].

Advancements in thin-film encapsulation (TFE) for bioelectronic implants emphasize the need for protective barriers against biological environments. Organic–inorganic multilayer TFEs stand out for their flexibility and biocompatibility, ensuring device integrity and functionality. Critical evaluation of their barrier and mechanical performance through water-vapor transmission rates is vital. This breakthrough in TFE technology is essential for the reliability and effectiveness of future implantable devices in healthcare and precision medicine [23]. Figure 1A–C shows the overview of implantable biosensors: applications, developments, and design concepts.

To address the issue regarding conformal contact between the implantable biosensors and the body, the device’s mechanical properties can be identical to the body, reflecting its softness, stretchability, and curvilinearity. However, there is still quite a disparity between flexible plastics and the human body, resulting in skin irritation and device failure due to movement-induced mechanical deformation. In contrast, textile-based sensors have mechanical properties like human skin, offering body-sensor-conform touch. Neural prosthetics, neural implants, devices for drug delivery, and diagnostic electronics all require minimally invasive materials. In this section, we will describe different materials used for implantable, biodegradable electronics and how they address the issues described above. 

### 2.1. Substrates and Insulators

#### 2.1.1. Paper

One of the oldest and most common natural origin ‘substrate’ materials is paper, made from plants’ cellulose. It is a mature substrate with attractive mechanical and surface properties, which makes it a suitable candidate as a base for electronics. Paper is lightweight, inexpensive, biocompatible, biodegradable, easily disposable, and annually renewable [26]. In addition, paper is flexible and can be folded or bent easily to form 3D structures without causing structural damage [27]. 

Recently, the use of paper as a substrate in electronic circuits has seen major applications such as the printing of arrays of organic field-effect transistors (OFETs) and OFET circuits on paper [28], printed electronics on banknotes to prevent counterfeiting applications [29], etc. Paper has been used for photovoltaic circuits [30] and disposable consumer products like thermo-chromic displays and electro-wetting displays [31].

The paper has also been used to develop biosensors named microfluidic analytical paper-based devices (μPAD) [32]. Paper-based sensors have shown excellent results in simultaneously determining glucose, lactate, and uric acid [33] and identifying heavy metal ions such as Cu^2+^ [34]. Hence, paper-based sensors are now competing against traditional sensors and offering a cheaper and simpler alternative. However, losing properties in aqueous environments and non-elasticity make paper-based devices unsuitable for applications where substrate elasticity plays an essential biochemical function as flexible devices.

#### 2.1.2. Silk

Silk is a natural fiber whose chemical structure can be expressed as a repeated sequence of three amino acids: glycine, alanine, and serine. The glycine content enables the beta sheets to be tightly packed and contributes to their compact structure and high tensile strength [35]. Other than its conventional uses in textiles, silk is an attractive substrate for developing electronics interfaced with living organisms because it is biocompatible [36], biodegradable [37] in both untreated and methanol-treated forms, bioresorbable [38], non-toxic, optically transparent [39], thin-film flexible [40], compatible with aqueous processing [41], and amenable to chemical and biological functionalization [42].

Because of its biocompatibility and controlled biodegradability (tunable from minutes to days), silk has been used as a medium for food-sensing electronics manufacturing [43], enhanced physiological monitoring, targeted drug delivery (especially in cancer care) [44], and as nanoparticles in the regeneration of peripheral nerves [45]. Silk has also been used as surgical sutures for bone and cartilage tissue engineering [46] because of its lower inflammatory response as well as being used as a substrate in biomedical implants for signal acquisition [47] and wireless activation of the therapeutic device [48] due to its conformal contact capability and non-invasive interaction with the smooth, curvilinear surface of biological tissues. Furthermore, recent research has shown that silk films can serve as a foundation for transistors [38] and a wide range of photonic devices [49].

#### 2.1.3. Gelatin and Shellac

Gelatin is another protein-based material that is biocompatible and biodegradable. The pharmaceutical industry uses it as a carrier capsule for oral drug delivery. In electronics, hard gelatin has been used as a substrate to build organic field effect transistor (OFET) devices on [11] and as coating of gelatin nanofibers with additional extracellular components or synthetic peptides, resulting in improved substrate flexibility and ease of adjustment for tissue regeneration applications and high throughput drug screening. The utilization of hydrogels derived from gelatin has yielded encouraging outcomes in the cultivation and implantation of tissue-engineered human corneal endothelial cells (HCEC) and in the functional regeneration of damaged soft tissue [50]. In skin tissue, gelatin/chondroitin sulfate nanofibrous substrates have demonstrated persistent chemical and physical support for cell growth due to wound healing stimulation [51]. 

An innovative microcapsule made from all-natural materials, specifically gelatin and shellac, these microcapsules are designed for bio-related applications, leveraging the biocompatibility and biodegradability of their constituent materials. This work represents a significant step forward in the development of environmentally friendly and sustainable materials made from gelatin and shellac for use in medical and pharmaceutical fields [52]. The utilization of gelatin and shellac in implantable biosensors represents a novel and developing field of study, where these materials have been applied in various aspects such as in the creation of coaxial fibers, surface modification processes, and as coatings on electrodes within biosensor systems [53,54,55].

Shellac is a naturally occurring polyester copolymer that consists of a complex blend of hydroxy acids, including both aliphatic and alicyclic varieties; historically, it has been used as a pigment, on furniture varnish decoration, as sealing wax, protective powder, and cotton dye. It possesses excellent smooth surface morphology and high solubility in alcoholic film-casting solvents, making it a suitable biodegradable substrate for high-performance organic electronic products such as OFET and inverters [56].

#### 2.1.4. Polymers (Synthetic Polymers)

Polymers, large molecules made up of smaller, recurring molecules called monomers, have recently received special interest as biomaterials due to their versatility in applications like prostheses, organ components, and hip and knee joints. Polymeric materials can be classified based on their natural, synthetic, and bioinspired source. Natural polymers like starch, cellulose, and collagen naturally occur in plants and animals. Synthetic polymers are artificial and developed through chemical processes. Bio-inspired polymers are made up of materials synthesized to mimic naturally occurring polymers. Natural polymers’ drawbacks include the potential for microbial contamination, antigenicity, and property variation from source to source. Synthetic polymers are more suitable as substrates in implants because of their quality, ease of processing, and manipulation flexibility (kinetics of degradation can all be regulated). Bioresorbable polymers offer a solution to the challenges associated with metal implants, including issues such as wear and debris formation, corrosion risks, and the need for implant removal, making them ideal materials for various trauma surgery instruments.

Collagen, a natural polymer, is known for its excellent biocompatibility, degradation, and interactions with cells and other macromolecules. Resorbable forms of collagen have been utilized to close graft and extraction sites, dress oral wounds, and promote healing [57]. Collagen-based membranes have applications in periodontal and implant therapy, while tissue-based collagen devices are extensively utilized in cardiovascular fields, including cardiac valves and vascular prostheses [58]. 

With a rise in interest in green electronics, synthetic polymers are becoming more important. For instance, Polydimethylsiloxane (PDMS) is a synthetic and transparent polymer, which is biocompatible, hemocompatible, and resistant to inflammation. Due to its low Young’s modulus, fluid impermeability, high dielectric strength, and chemical resistance, PDMS has emerged as the preferred synthetic substrate for creating electronic platforms compatible with living organisms. Microelectrode arrays embedded within PDMS microchannels have been developed to monitor the bladder afferent nerve activity [59].

PDMS has also been employed in implant prosthetic surgeries by constructing microfluidic devices and evaluating biomaterials for in vivo and in vitro testing [60]. Given its excellent properties, PDMS has the potential for applications in treating internal organs via living tissue implants and in stretchable electronics (as a substrate to join organic and inorganic devices through interconnected stretchable electrodes), optoelectronics, and integrated systems [61,62]. In addition to PDMS, alternative synthetic materials are presently utilized in the manufacturing of implantable devices, such as polylactic acid (PLA), polylactic-co-glycolic acid (PLGA), and polyvinyl alcohol (PVA). Although PLA itself may not be the most suitable choice for a substrate due to its limited processing temperature and low glass transition temperature (58 °C), PLA can be transformed into a more usable form by using additives or blending it with other polymers, which can eventually be used to manufacture bioresorbable scaffolds for growing living cells [63]. PLA-based substrates do not pose toxicity concerns, which is why polymers derived from lactic and glycolic acids have been widely used in the construction of biodegradable sutures and medical devices [64]. PLGA is also a bioresorbable substrate that has seen its use in drug delivery systems because of its ability to manipulate the degradation in the human body and in medical implants [65].

Chitosan, a natural polymer derived from chitin found in the shells of crustaceans, offers remarkable biocompatibility, biodegradability, and non-toxicity, making it an attractive material for biomedical applications. Its unique properties have facilitated its use in wound healing applications, drug delivery systems, and as a scaffold material for tissue engineering. Chitosan’s ability to form hydrogels is particularly valuable in creating scaffolds that support cell growth and tissue regeneration [66]. Alginate is another naturally occurring biopolymer extracted from brown seaweed, and it is widely recognized for its gel-forming capabilities, which have been exploited in drug delivery systems, wound dressings, and tissue engineering. Alginate’s versatility and mild gelation conditions are conducive to encapsulating a wide range of biological materials, including cells and therapeutic agents, without compromising their viability or activity [67]. Polyhydroxyalkanoates (PHAs) are a family of biodegradable polymers produced by microbial fermentation processes. Their biocompatibility and biodegradability, coupled with their physical and mechanical properties, make them suitable for various medical applications, including sutures, bone plates, and drug delivery systems. PHAs’ tunable degradation rates are particularly beneficial for developing long-term implantable devices [68].

Hyaluronic acid, a natural polymer present in the human body, plays a crucial role in tissue hydration, lubrication, and repair. Its biocompatibility and biodegradability have led to its widespread use in ophthalmic surgery, dermal fillers, and as a carrier in drug delivery systems. Additionally, hyaluronic acid-based hydrogels are used in tissue engineering to provide a hydrated, 3D environment conducive to cell growth and differentiation [69]. Silk fibroin, derived from the cocoons of silkworms, is a protein-based natural polymer known for its exceptional mechanical strength, biocompatibility, and controlled degradation. Silk fibroin has been used in various biomedical applications, including sutures, tissue engineering scaffolds, and drug delivery systems. Its ability to be processed into various forms, such as films, gels, and fibers, adds to its versatility in medical applications [70].

Lastly, PVA, a synthetic polymer, has recently found application as a substrate in biodegradable and biocompatible electronics production. It is a water-soluble polymer that forms flexible layers. When utilized as a substrate or coating for implantable devices, PVA facilitates the controlled movement of water-soluble analytes by modifying the density of crosslinking between its chains and their ability to swell. Advanced electronic components, such as photodetectors, transistors, light-emitting diodes, rectifying diodes, and sensors, have been successfully manufactured on a sacrificial PVA substrate. These devices are designed for physiological measurement and stimulation through the human epidermis [71]. The soluble PVA layer acts as a temporary support, allowing for the transportation and mounting of the device onto the skin. Later, it can be easily removed by rinsing with water, ensuring a non-permanent attachment.

Among these, PDMS is mainly used in implantable biosensors, biomedical device coatings, and graphene electrode integration; PLA is applied in multilayer microneedle fabrication and drug-eluting coatings; PVA features in hydrogel composites for drug delivery and hydrogel electrolytes for implantable sensors [72,73,74,75,76,77].

### 2.2. Active Layer

#### 2.2.1. Inorganic Semiconductors

Silicon (Si) is the primary inorganic material utilized in the semiconductor industry. Although silicon is known to be firm and stiff, in thin layers, it can withstand the changes while measuring the fluctuations in arterial blood pressure when it is used as a membrane in pressure sensors [78]. Single-crystalline silicon nanomembranes (Si NMs) undergo complete hydrolysis, which makes Si an ideal candidate for active, biodegradable electronic implants [79]. Silicon derivatives, such as silicon oxide and single-crystal quartz have found purpose in implantable blood pressure measurement surface acoustic wave sensors [80]. Among the Si alloys, silicon nitride, Si_3_N_4_ in its most thermally stable form, displays promise in bio-MEMs orthopedic sensors due to its biocompatibility with human bone cells in vitro [81].

Metal oxide semiconductors have also been studied for biomedical applications. Among these, TiO_2_ and Zn(OH)_2_ (a by-product of ZnO_2_) are non-toxic to humans [82]. However, these are unfavored due to the high cost of processing and their incompatibility with biodegradable substrate materials [83]. The inorganic semiconductors discussed above may not be favorable due to their incompatibility with the body, requiring encapsulation in materials like silicone and perylene adding to the convolution of the device. These materials may cause stress and multimodal deformation due to their high stiffness and pose serious hazards to the body due to the existing sharp edges, forgetting the unknown long-term effects on the artery and tissue [35]. 

#### 2.2.2. Organic Semiconductors

Organic semiconductors are organic materials with electrical conductivity between insulators and metals, which are placed as active layers to replace the traditional silicon-based devices. With their mechanical flexibility, organic semiconductors offer greater advantages in comparison to inorganic counterparts by allowing the fabrication of devices that are not only thin but stretchable. Organic stretchable electronics are lightweight, less costly, allow large-scale solution manufacturing, promote fine-tuning through molecular customization techniques, and provide the ability to withstand cracking, creasing, and folding. 

**Figure 2:**
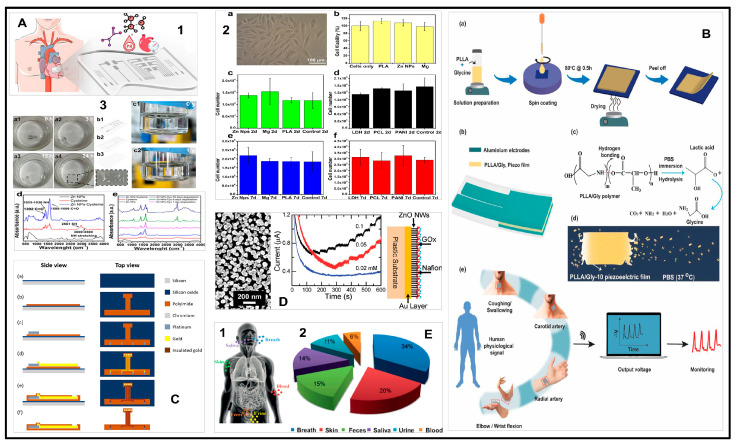
(**A1**) Concept of implantable multifunctional sensors for cardiac monitoring. (**A2**) Biocompatibility and cytotoxicity tests of cardiac monitoring sensor. (**A2a**) Shape and morphology of the H9c2(2–1) cardiac cells model. (**A2b**) MTT cytotoxicity results. (**A2c**) Cell number of the constituent materials after 2 days. (**A2d**) Cell number of the sensing materials after 2 days compared to the control. (**A2e**) Cell number of the constituent materials after 7 days. (**A2f**) Cell number of the sensing materials after 7 days. Degradation results of the Mg electrode in SBF at 0, 3, 12, and 24 h accordingly. (**A3a1**–**A3b3**) Schematic of the degradation of the Mg electrode in SBF. (**A3c1**) Beginning of the degradation of PLA. (**A3c2**) Degradation of the PLA substrate in SBF after 1 year. Fourier transform infrared (FTIR) diagram of (A3**d**) the Zn NPs, a thiol, and their combination. (A3**e**) Degradation of the Zn NP-thiol in SBF over time. Adapted in part with permission from ref. [84] under the terms of the Creative Commons Attribution CC by 4.0 (copyright 2021 American Chemical Society) (**Ba**) Schematic demonstration of the fabrication process for biodegradable piezoelectric PLLA/Gly film. (**Bb**) Schematic illustration of self-powered wearable sensor based on biodegradable piezoelectric film. (**Bc**) Disintegration of PLLA/Gly polymer due to hydrolysis when immersed in PBS solution. (**Bd**) Schematic illustration of PLLA/Gly piezoelectric film degradation in PBS solution at 37 °C for 5 days. (**Be**) Schematic demonstration of the real-time applications of the flexible piezoelectric sensor in healthcare monitoring. Adapted with permission from ref. [85], Copyright 21 March 2024 (Wiley Advanced Materials Technology). (**C**) A dental implantable temperature sensor on a flexible polyimide film. (**Ca**) Deposition of a silicon oxide sacrificial layer on a 100 mm bare silicon wafer. (**Cb**) Deposition of the first polyimide layer. Micropatterning of (**Cc**) the Cr/Pt layer for a temperature sensor and (**Cd**) the Cr/Au layer for both interconnection lines and pads. (**Ce**) Deposition of a second polyimide layer as an insulation layer, maintaining contact openings by photolithography. (**Cf**) Extracted sensor from the silicon wafer. Adapted with permission from ref. [86] under the terms of the Creative Commons Attribution CC by 4.0 Copyright MDPI Sensors 2020. (**D**) Flexible enzymatic glucose biosensor based on ZnO nanowires supported on a gold-coated polyester substrate. Adapted with permission from ref. [87], Copyright 2010 American Chemical Society. (**E1**) Different biofluids of volatile organic compounds (VOC) in the human body. (**E2**) VOC percentages in different biofluids following the data of healthy humans. Adapted with permission from ref. [88], Copyright 2018 American Chemical Society.

The polymer backbone determines the semiconducting properties of polymers, specifically its p-conjugation. Natural p-conjugated molecules include carotenoids (e.g., b-carotene and bixin) or even melanin that can become conductive with doping through water absorption. In melanin, that acquired conductivity comes from electron and proton conduction, leaving it to a wider range of applications in ionic conduction and consequently as an excellent candidate for bioelectronics interfacing, e.g., neuronal cell coupling [35]. Another example of naturally occurring small molecules capable of substantial p-p stacking is dye molecules, known for their range of colors. For example, in the indigo molecules, due to their strong intermolecular hydrogen bonding, p-stacking is strengthened along the crystallographic b-axis, leaving the molecules with excellent anisotropic charge transport properties. Another naturally occurring molecule is peptide nanostructures (PNSs). Known for their biodegradability and promise in drug delivery systems and sensors, recent research follows the functionalization of PNSs with blue-emitting conjugating polymers to self-assemble LED with an 80% biodegradability via enzymatic action. Figure 2A–E presents a comprehensive examination of implantable sensors for heart monitoring, materials’ toxicity and breakdown, and continuous health surveillance applications.

With the onset of an impressive electric charge transport execution by a multitude of H-bonded semiconductors that do not possess a p-conjugated backbone, the next step that should be investigated is the reassessment of H-bonded individual nucleobases and nucleobase pairs. A new field of DNA research could be accessed through the meticulous optimization of advantageous orientations of these molecules.

#### 2.2.3. Integration of Transistors

Transistors, whether inorganic or organic, are pivotal in signal transduction and amplification within bioelectronic devices, ensuring sensitive, accurate physiological monitoring while maintaining compatibility with biological systems [89,90]. Integration of semiconductors into bioelectronic devices critically relies on transistors, essential for controlling and modulating electronic signals. Silicon-based transistors, utilized in inorganic semiconductor devices, are foundational in applications such as pressure sensors for amplifying physiological signal changes [91]. Their fabrication processes and reliability make them a staple in bioelectronics [92].

Organic field-effect transistors (OFETs) represent a significant leap in using organic semiconductors, offering flexibility and biocompatibility conducive to biological tissue interfacing [93]. OFETs are particularly suited for dynamic applications like neuronal cell coupling, thanks to their mechanical properties that accommodate bending and stretching without compromising functionality [94,95]. The structure of organic field-effect transistors (OFETs) and their integration into implantable and biodegradable sensors is a complex and evolving area of research. OFETs, which utilize organic semiconducting layers, have been shown to offer compatibility with flexible and biodegradable substrates, making them ideal for a wide range of applications, including wearable and implantable electronics. Key research has demonstrated the use of pentacene and other π-conjugated polymers within OFETs to achieve devices that are not only biocompatible and biodegradable but also capable of maintaining high performance over multiple bending cycles. This is crucial for the development of sensors that can be used in medical diagnostics and environmental monitoring before they naturally degrade. Recent progress emphasizes polymer dielectrics like silk fibroin in OFETs for enhanced performance, biodegradability, and flexibility, showcasing the benefits of bio-derived materials for eco-friendly device functionality. These insights reveal OFETs’ promise for sustainable sensor tech, merging organic electronic benefits with eco-friendly demands [96,97,98].

### 2.3. Dielectrics

Dielectric materials, which function as electrical insulators and can undergo polarization under the influence of an electric field, have attracted significant attention in recent times. Biodegradable materials such as dielectrics have gained interest. For instance, biodegradable Deoxyribonucleic acid (DNA) has been employed as electron-blocking layers in organic light-emitting diodes (OLEDs) and as gate dielectrics in OFETs [99]. DNA has also been employed in developing nonlinear optoelectronic modulators and photonic arrays. The nitrogenous bases of DNA: adenine, guanine, thymine, and cytosine, show promise for low operating voltage (~0.5 V) OFETS as gate dielectrics with large breakdown fields of approximately 1 MV/cm to approximately 3.5 MV/cm [11]. 

Another notable dielectric material is albumen, which is found in chicken egg whites. The gate dielectrics of flexible OFETs and the complementary inverters are prepared by the thermal treatment of the albumen [100]. Chicken albumen, a cost-effective biomaterial, for constructing microlasers holds significant promise for medical and biosensing applications. Fabrication was performed for rhodamine B-doped chicken albumen microspheres, varying in size from 20 μm to 100 μm through an efficient emulsion process. These microspheres exhibited lasing emission under optical pulse excitation, attributed to the whispering gallery mode (WGM), with a notable threshold of 23.2 μJ mm−2 and a high Q-factor of approximately 2400 in an 82 μm diameter microsphere. The size-dependent lasing characteristics of these albumen-based bio lasers align with the WGM theory, and their functionality in aqueous and biological environments like water and human blood serum underscores their potential in biosensing and biological applications [101]. Egg albumen is also an effective dielectric material for developing memristor devices and these devices are made with water-soluble egg albumen and dissolvable magnesium and tungsten electrodes. These devices exhibit stable bipolar resistive switching behavior. The research demonstrates the potential of using natural, biocompatible materials like egg albumen in bioelectronics and environmental sensors, highlighting its advantages in terms of biodegradability and environmental friendliness [102].

Members of the sugar class: lactose, glucose, and sucrose are also good candidates for natural dielectrics because of their low dielectric loss (10^−1^ at 10 mHz for glucose). Their ease of processing in aqueous solvents makes them more advantageous for applications such as in OFETs [11]. Cellulose also demonstrates promise as a gate dielectric material in organic thin-film transistor (OTFT) devices. Cellulose-based material used as a dielectric gate in OTFTs, and complementary inverter circuits exhibits high DC gain (over 500 V) and wide noise margins (up to 92.5%), ensuring that the inverter device’s output voltage signal is free of interference, allowing optimum response and efficiency [103]. Biodegradable synthetic polymers such as PLA, PVA, and PDMS are also applicable as dielectric layers in OTFTs [104].

### 2.4. Electrodes and Interconnects

Electrodes serve as electrical conductors that facilitate the movement of charged carriers between the system and the external circuit. The use of organic polymer-based electrode materials is gaining recognition due to their ability to exhibit satisfactory electronic conductivity when doped, as well as their capability for both electronic and ionic conduction [105]. Furthermore, polymers offer superior mechanical flexibility compared to metals, making them well-suited for the fabrication of flexible electronics. Melanin and PEDOT are two examples of polymer electrodes that have been utilized in unique applications, including ion bipolar junction transistors, organic electronic ion pumps, and in vivo electrocorticography (ECoG) measurements from mouse brains [106].

Melanin is a natural biopolymer found in various biological systems and it is increasingly being explored for its potential applications in biosensors. Recent studies have shown that melanin can be used effectively in Extended Gate Field Effect Transistors (EGFETs) as an active layer for pH sensing. These melanin-based EGFETs have sensitivities ranging from 31.3 mV/pH to 48.9 mV/pH. This sensitivity is attributed to specific binding sites in melanin’s structure, such as hydroxyl groups and quinone imine, which interact with H+ ions in solutions [107].

Moreover, melanin is considered advantageous for bioelectronic applications because of its biocompatibility. This makes it a promising material for developing electronic devices that interface with biological systems, such as brain neurons. However, challenges exist in using melanin for these applications, primarily due to its complex synthesis process and difficulty in dispersing in an aqueous medium. Recent advancements have addressed these issues, allowing for biosynthetic melanin production that resembles natural melanin, which can be synthesized in a few hours with enhanced solubility and homogeneity. This breakthrough facilitates the production of high-quality melanin films for use in bioelectronic devices, such as transistors, electrical contacts, pH sensors, and photovoltaic cells [108].

On the other hand, PEDOT: PSS (poly(3,4-ethylenedioxythiophene): poly (styrene sulfonate)) is widely utilized in biosensors for its high conductivity, biocompatibility, and stability. It is particularly useful for making soft bioelectronics for its property of electrically coupling with tissues for sensing and stimulation. PEDOT: PSS hydrogels, for example, have been developed with high conductivity and biocompatibility and they are suitable for in situ electrochemical sensors within 3D cell cultures. These characteristics make PEDOT: PSS a versatile material for bioelectronic applications and the development of biosensors that require biocompatible environments, high transconductance values, and low operational voltages [109].

Next, because they are resistant to corrosion and do not react, nontoxic metals like titanium (Ti) can be used in the medical field for bone and dental implants. Gold and silver have already been used in the manufacturing process for dental fillings. Since these metals are typically resistant to breaking down, accumulation and inevitable blockage in the body may be caused if employed in excess. To combat this, other physiologically friendly metals have been investigated like manganese (Mn), zinc (Zn), and magnesium (Mg). Any remaining metabolites can be excreted from the body or harmlessly absorbed. In the case of magnesium, magnesium purification, anodized coatings, and selective alloying are effective techniques to decrease the degradation rate in the human fluid environment. 

To gain a thorough understanding of implantable devices, it is crucial to incorporate layers or materials that possess intermediate functionality. One such element is the interconnect layer. Biocompatible polymer polyurethane has been used to develop water-based isotropically conductive adhesives (ICAs) for this purpose. These waterborne polyurethane ICAs offer a viable alternative to the conventional oil-based versions. The rheological properties of water-based ICAs are compatible with various high-throughput printing techniques, including screen printing and roll-to-roll printing. This makes them suitable for applications such as electrical interconnects and low-cost printed circuits [110]. 

## 3. Biodegradation Mechanisms

Biodegradation is paramount for implantable sensors as it ensures they safely break down post-use, avoiding the need for additional surgical removal, reducing patients’ risk, and lessening healthcare costs. Biodegradable materials also prevent long-term adverse bodily reactions and align with eco-conscious practices by minimizing environmental waste. This attribute is integral for advancing personalized medicine, allowing for temporary monitoring without lasting bodily impact, and upholding ethical medical standards focused on patient safety and sustainability. Biodegradation thus stands as a cornerstone in the responsible evolution of implantable medical devices. 

### 3.1. Polymers

**Figure 3:**
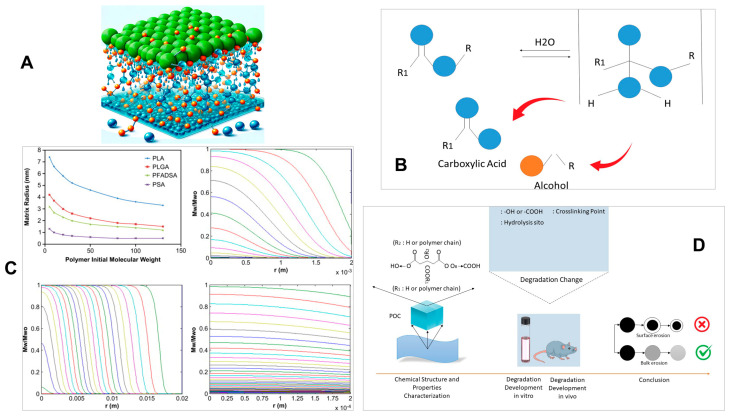
(**A**) Degradation of polymer matrix response to the presence of water; blue molecules are unbonded, orange molecules are loosely bonded, and green molecules are strongly bonded. (**B**) The hydrolysis reaction of water with susceptible bonds of a compound forms two or more products. (**C**) Degradation profiles of four different polymer types (PLA, PLGA, PFADSA, PSA), reprinted from *The Lancet*, ref. [113], Copyright (2009), with permission from Elsevier. (**D**) Systematic representation of in vivo and in vitro degradation behaviors of poly(1,8-octanediol-*co*-citrate) (POC), density decline with degradation, mass loss, and PBS–absorption ratio provoke nonlinearly, the morphology changes and the descent of mechanical properties due to degradation.

Polymer degradation can occur through two distinct mechanisms: surface erosion and bulk degradation. The critical factors influencing the degradation of a polymer matrix are the size of the matrix, the rate at which bonds are cleaved, and the ability of water or enzymes to diffuse within the matrix [111]. Surface erosion is limited to the outer surface of the implant, while bulk degradation affects the entire implant [112]. Consequently, the mechanical strength and molecular weight of polymers gradually decreased over time, causing the implant to disintegrate and produce polymer debris. However, in the short term, the mechanical strength, original shape, and molecular weight remain relatively stable, enabling the polymers to effectively protect sensors as a cohesive unit. Figure 3 illustrates polymer degradation in water, hydrolysis reactions, profiles of various polymers (PLA, PLGA, PFADSA, PSA), and detailed degradation behaviors of POC, showcasing mass loss, absorption ratios, and mechanical property changes.

The in vivo degradation of poly (α-hydroxy acids) such as PLA and polyglycolide primarily occurs through hydrolysis, enzymatic degradation, and oxidation processes [114]. Initially, the ester bonds in the polymer chains undergo random hydrolytic scission, resulting in the fragmentation of the polymer into oligomers and monomers such as lactic acid, glycolic acid, and 6-hydroxyhexanoic acid. Eventually, these monomers metabolize carbon dioxide and water [115]. Various environmental factors, including the site of implantation and mechanical stress, can influence the hydrolysis of polymers, along with their chemical composition, molecular weight, monomer concentration, porosity, and volume-to-surface area ratio. Amorphous copolymers like PLGA and P(L/DL) LA degrade gradually. In a clinical setting, the absorption of PLGA implants typically takes around 1 to 1.5 years, P(L/DL) LA implants take 2 to 3 years, and PLLA implants may require 5 years or more for complete absorption in a biological organism [116]. 

Unlike poly (α-hydroxy acids), PVA exhibits stability within the body [117], yet it can be readily absorbed by the body due to its high water solubility. When used as artificial cartilage, PVA hydrogel has been observed to remain intact for 2 years within the knee cartilage in vivo. PVA with a low molecular weight is eliminated through the kidneys, while PVA with a high molecular weight accumulates in the spleen and liver, where it may persist for up to 90 days before being excreted through urine [117]. Recent advancements have focused on the development of novel PVA-based biomaterials with intelligent characteristics such as shape memory and injectability, which will be valuable for the creation of injectable electronics [118]. 

PGS, another polymer approved by the FDA, undergoes in vivo degradation primarily through surface erosion caused by the hydrolytic cleavage of cross-links within the elastomer [119]. Throughout the degradation process, PGS maintains its structural integrity, allowing it to retain its original geometry, which is crucial for certain applications [120]. Non-porous PGS experiences a 70% decrease in mass over 35 days and is completely absorbed within 60 days. The degradation rate of PGS in vivo ranges from 0.2 to 1.5 mm per month [121], and this rate can be adjusted by altering the degree of cross-linking and porosity. During degradation, PGS breaks down into glycerol, which is a fundamental building block of lipids, and sebacic acid. Both degradation products are naturally metabolized, as sebacic acid is a metabolic intermediate in fatty acid oxidation. 

To prolong the operational lifespan of implantable sensors, their components can be encapsulated in polymers like PHB and POMaC, which have a higher durability in bodily fluids. PHB is a member of the polyhydroxyalkanoates family, which consists of synthetic biodegradable polyesters [122]. In the human body, PHB undergoes degradation through enzymatic and non-enzymatic reactions, resulting in the release of hydroxybutyric acid (3HB), a normal metabolite in the bloodstream. The degradation rate of PHB can be modulated by factors such as macrophages, copolymerization, and blending natural isotactic PHB with synthetic atactic PHB [123]. On the other hand, POMaC hydrolytically degrades into its fundamental constituents known as poly (diol citrates) (POCs), which have already demonstrated biodegradability [124]. The degradation rate of POMaC can be tuned by varying the molar ratio of maleic anhydride and the degree of cross-linking. Complete degradation of POMaC has been observed both in vitro and in vivo, with an in vivo degradation duration of up to five weeks [125]. 

### 3.2. Silicon-Based Materials

Bulk Si is usually non-biodegradable due to the formation of native oxides on its surface. A nanoform of it, however, can be completely dissolved in biological fluids [126]. Both Si and SiO_2_ degrade into silicic acid under physiological conditions. The rate of degradation is influenced by factors such as pH, temperature, and protein concentration, and the degradation rate of Si can be adjusted by doping it with other substances [127]. The degradation of Si occurs through its oxidation to SiO_2_ or by direct equilibration, as depicted by the following reactions:

Si + 4H_2_O ⟶ Si(OH)_4_ + 2H_2_ (1)

SiO_2_ + 2H_2_O ⟶ Si(OH)_4_ (2)

The rate of dissolution remains constant and shows no dependence on thickness, approximately 10 nm per day, while the surface maintains its smoothness without any cracks or particulates [19]. When lightly doped silicon nanostructures (Si-NMs) are used, they undergo complete hydrolysis during the process, resulting in safe degradation products. However, other Si-based materials such as SiO_2_ and Si_3_N_4_, commonly used as substrates or insulators, exhibit a slower degradation rate of around 8 nm per day. The generation of hydrogen gas during Si degradation can potentially harm surrounding tissues, whereas the primary degradation product of Si-based materials, silicic acid, can be eliminated in small quantities by the liver, spleen, and lungs, ultimately being excreted by the kidneys [128].

### 3.3. Metals 

The biodegradable metals degrade into metal cations via hydrolysis; the body subsequently absorbs the byproducts of ionic degradation. Biodegradable metals vary in dissolution kinetics and advantages, making them suitable for different applications. Metal selection is affected by a variety of factors, such as the specific functionality of the device, its placement, its anticipated function in the body, and its physiologically permissible concentration limits.

Due to its excellent biocompatibility and conventional fabrication techniques, magnesium (Mg) is the most used alkaline metal. There are two mechanisms by which Mg degrades: electrochemically and mechanically. Electrochemical degradation of Mg results in magnesium oxide (MgO) being produced at the degradation surface. Hydrolysis converts MgO to magnesium hydroxide Mg(OH)_2_ according to the following reaction, which is subsequently metabolized by the body [129]:

MgO + H_2_O ⟶ Mg(OH)_2_ (3)

In addition, Mg can become an ion when it loses electrons, resulting in Mg hydroxide and hydrogen being created:

Mg ⟶ Mg^2+^ + 2e^−^ (4)

H_2_O + 2e^−^ ⟶ H_2_ + 2OH^−^ (5)

Mg^2+^ + 2OH^−^ ⟶ Mg(OH)_2_^−^ (6)

As the degradation progresses, Mg(OH)_2_ is precipitated on the metal surface, but chloride ions may dissolve it further. Furthermore, the degradation products increase the local pH and alter the Mg^2+^ concentration around the implant [129]. Even though complex physiological and chemical reactions occur around an implant, the Mg(OH)_2_ layer deposited on metal surfaces slows the degradation rate of Mg, thus preventing a continuous increase in metal ions, H_2_, and pH during their degradation [130]. Since Mg is required for human metabolism, it does not cause cellular toxicity in the body. However, the pace of breakdown should be regulated due to the toxicity owing to hydrogen gas accumulation. 

The fast dissolution rate and non-uniform degradation of alkaline metals like Mg in vivo make transition metals a better alternative for applications requiring long-term stability [128]. Transition metals can survive in biofluids for a sizable amount of time even without encapsulation. As an example, the transition metal Mo degrades in the body by hydrolysis into molybdenum dioxide (MoO_2_):

Mo+2H_2_O ⟶ MoO_2_ + 4H^+^ + 4e^−^ (7)

Among the surface oxides of Mo, MoO_2_, which is prominent in the pH range of the body, determines its degradation behavior. Following the formation of oxides, Mo and MoO_2_ are simultaneously dissolved, with the oxide dissolving more slowly. Because of cracks on their surface, oxide layers do not hinder dissolution [131]. Degradation rates range between 0.25 and 15 μm per year. Encapsulation in a polymer can prolong the lifetime of the material from a few hours to days, depending on the potential applied and the local concentration [132].

Zn is also a transition metal, but its surface does not degrade uniformly, unlike those previously discussed. In aqueous solutions, zinc oxide (ZnO) and zinc(OH)_2_ are the dominant surface products. It is estimated that Zn oxide dissolves at 120–170 day^−1^, whereas bulk Zn degrades at a rate of 0.4 mg (day·cm^2^)^−1^ [133]. Due to the possibility of adverse immune responses caused by the degradation products of biocompatible metals, the local concentration of these materials should be kept below a threshold. In implant applications requiring more metal mass, such as electrode arrays and mechanical supports, alloys may be preferable to pure metals because of their slower and more controlled degradation [134]. Additionally, alloys possess a higher mechanical strength than pure metals. For instance, a decrease in degradation rate is observed for Z_n−x_Cu alloys as the Cu concentration in the alloy increases. Z_n−x_Cu alloys degrade slowly in simulated bodily fluid solutions, with rates ranging from 22.1 ± 4.7 to 33.0 ± 1.0 μm/year [119]. 

In vitro testing on human endothelial cells has shown an acceptable level of cytotoxicity for Z_n−x_Cu alloys. A biodegradable implant can benefit from these alloys because they combine strength, ductility, and antibacterial properties. When Aluminum (Al) is added to Zn alloys, their degradation rate is slowed, and their hardness is increased. Additionally, Mg-Al alloys demonstrate low cytotoxicity and possess an elastic modulus comparable to human bone [135]. Therefore, the benefits of these metals are combined when Zn, Al, and Mg are combined to form a ternary Zn-Al-Mg alloy. This alloy’s degradation rate can be tuned by altering the Mg mass [135]. In addition to the appropriate alloying, the degradation rate of metals can be reduced by controlling their microstructure, i.e., their texture and grain size, and by surface modifications or coatings. Any excess intake of magnesium (Mg), molybdenum (Mo), and zinc (Zn) and their degradation products are eliminated from the body via the kidneys.

## 4. Sensing Mechanisms of Biodegradable Biosensors

The mechanisms of biodegradable sensors are diversified into the following variations of different physical properties, such as capacitance, resistivity, triboelectricity, and piezoelectricity [111]. This section will discuss their sensing principles and touch base on their applications. 

### 4.1. Resistive Sensors

Resistive sensors are built upon the piezoresistive effect. A piezoresistive effect is caused by material structural deformation resulting in a change in resistance. This change is exhibited as an electric current [136]. Usually, the gauge factor (GF) is used to represent the sensitivity and it is expressed as follows:

GF = (ΔR/R_0_)/ε^−^ (8)

where ΔR represents the change in resistance, R_0_ represents the initial resistance, and ε stands for the strain/pressure [137]. Usually, a high GF endows the device with high sensitivity, whereas physiological activities that are weak but significant may result in minor changes in R, resulting in low GF values. It is often the case that semiconductors exhibit an excellent piezoresistive effect and are high in sensitivity, making them an excellent choice for piezoresistive sensors. A metallic SWNT displayed a remarkable relative differential resistance sensitivity of 27.5% per nanometer, alongside a piezoresistive gauge factor reaching up to 2900 [138]. It is important to note, however, that GF values can be sacrificed for stretchability, suggesting there is a trade-off between the two, especially with skin-mounted devices. Si nanomembrane (Si-NMs)-based strain gauges, for example, offer a piezoresistive response in bending strains in a bioresorbable pressure monitoring platform for continuous monitoring of intracranial space pressure due to differences between the pressure of air trapped inside the cavity and the surrounding environment [139]. A monocrystalline flexible silicon sheet serves as the device’s encapsulation layer that resists biofluid penetration and resorbs at a controlled rate. Piezoresistive materials are also commonly used for temperature sensors, where they take advantage of the resistance dependent on temperature. In these sensors, two kinds of behaviors are observed: their resistance increases with increasing temperature (positive temperature coefficient, PTC) or decreases with increasing temperature (negative temperature coefficient, NTC). It is therefore possible to use sensors with PTC behavior for self-regulating heaters, overcurrent protection materials, and microswitches. In contrast, sensors with NTC behavior could be used for temperature measurement, mapping, and compensation in highly stretchable thermistors. Lightweight, biodegradable CB/CPPC foams with a precise closed-cell structure were developed through melt blending and chemical foaming, significantly reducing the electrical percolation threshold from 2.48 vol% to 0.138 vol%. These foams exhibit a sensitive, nearly linear negative temperature coefficient (NTC) effect from 25 °C to 70 °C, ideal for wearable electronics and temperature sensors in various applications, demonstrating a novel approach for creating NTC materials [140]. Resistive temperature sensors that are completely biodegradable and highly formable have been employed in medical post-surgery monitoring. A study introduces biodegradable temperature sensors with rapid response times of 10 ms and consistent performance under mechanical stress, featuring less than 0.7% resistance variation when deformed. Encapsulated in a compostable polymer that mimics the mechanical properties of muscle and cartilage, these sensors, when organized into arrays, offer a sustainable solution for flow mapping and potential applications in food safety and post-surgery patient monitoring, enabling wireless operation with a 200 mK resolution [141]. 

Another study introduces an implantable sensor made from entirely biodegradable materials, designed for real-time monitoring of tendon stress post-surgery. Featuring dual sensors for independent strain (down to 0.4%) and pressure (as low as 12 Pa) measurement without interference, the device boasts quick response times, minimal hysteresis, and enhanced cycling stability due to an optimized biodegradable elastomer, showing a 54% performance improvement. Demonstrated biocompatibility and functionality in a rat model highlight its potential for facilitating personalized rehabilitation by monitoring tendon healing, aiming to eliminate the need for device removal surgery [142].

### 4.2. Capacitive Sensors

Capacitive sensors offer unique advantages over resistance-type sensors due to their higher linearity, low hysteresis, and thermal stability [137]. Typically, capacitive sensors consist of two electrodes sandwiched between a dielectric layer. The capacitance value is determined by the following equation:

C = εA/d^−^ (9)

where ε is the dielectric constant, A stands for the plate area, and d represents the distance between the two parallel plates. Capacitive sensors behave similarly to resistive sensors in terms of their sensitivities.

S = (ΔC/C_0_)/P (10)

where ΔC represents the change in capacitance, C_0_ represents the initial capacitance, and P stands for the applied pressure. The model’s sensitivity strongly depends on ΔC, as can be seen from the equation.

Biodegradable pressure sensors with capacitive structures, i.e., those utilizing air or biodegradable dielectric materials, or piezocapacitors, have been developed for health monitoring. These devices are implanted in various parts of the body to prevent dangerous intracranial pressure in organs like the brain, eyes, or muscles following surgery. Research introduces advanced bioresorbable pressure sensors with significantly extended lifetimes, capable of accurate intracranial pressure monitoring in rats for 25 days, surpassing current devices by tenfold. These sensors minimize surgical risks, costs, and patient discomfort while proving their biodegradability and clinical utility through comprehensive safety assessments [128]. An electrodeposited Zn/Fe parallel plate containing air and connected to a microfabricated inductor coil is one example of a biodegradable wireless capacitive pressure sensor based on the resonant frequency mechanism. Tested wirelessly in air and saline, the sensor displayed a linear response to pressure, showing a sensitivity shift from 39 kHz/kPa in 0–20 kPa range to stabilizing at −54 ± 4 kHz/kPa after 20 h in saline, remaining operational for 86 h [133]. By applying pressure to the sensor, the gap in the capacitive structure is reduced and the resonance frequency is shifted, which can be measured wirelessly by an external coil. In response to applied pressure, the fabricated sensor displayed a linear behavior, and the sensor sensitivity in the 0−20 kPa pressure range was approximately 290 kHz kPa^−1^. 107 h of functional life was observed for the sensor in a saline solution, followed by 170 h of complete degradation. 

The innovative microneedle biosensor with an interdigitated electrode (MAIDE) for in situ capacitive detection of proteins showed promising performance, capable of detecting bovine serum albumin (BSA) concentrations down to 21 ng/mL across ranges of 100, 10, and 1 µg/mL. It demonstrated stable capacitance readings in vivo with less than 0.5% deviation, and satisfactory biodegradability within 10 h, indicating its potential for biodegradable and wearable/implantable capacitive biosensing applications [143]. 

### 4.3. Piezoelectric Sensors

Piezoelectricity is unique in that it can convert mechanical or vibrational energy into electrical energy and the other way around. Inorganic ceramics and organic polymers are the main piezoelectric materials used to develop new generations of piezoelectric nanogenerators. A high electric field or stretching causes molecular dipoles in organic polymers to reorient, giving rise to the piezoelectric effect. The sensor achieved high efficiency in generating electrical output, reaching approximately 200 V and 150 μA·cm^2^ during bending motions, and was able to power over 100 blue LEDs directly from human finger movements without any external source, demonstrating superior performance compared to existing flexible piezoelectric generators [144]. Poly(L-lactic acid) (PLLA), PVDF, and poly(D-lactic acid) (PDLA) are the most common organic piezoelectric materials [145]. The implantable biodegradable piezoelectric sensor, made from Poly-l-lactide (PLLA) and approved medical materials, accurately measures pressures ranging from 0 to 18 kPa and maintains reliable performance for up to 4 days in aqueous environments. Demonstrated in vivo within a mouse’s abdominal cavity to monitor diaphragmatic pressure, this sensor presents a promising solution for intraorgan pressure monitoring, with significant potential for applications in regenerative medicine, drug delivery, and medical devices [146]. For inorganic materials, piezoelectric potentials can be formed by altering the ion balance when anions and cations are displaced relative to one another, thus converting mechanical energy to electrical energy. Aluminum nitride (AlN), zinc oxide (ZnO), barium titanate (BaTiO_3_), lead zirconate titanate (PZT), lithium niobate (LiNbO_3_), and quartz are the most studied inorganic piezoelectric materials [147,148,149,150,151]. Inorganic ceramics have been shown to have significant piezoelectric capability. However, they cannot be directly integrated into flexible devices because of their intrinsic fragility. On the contrary, when compared to inorganic ceramics, organic polymers have intrinsically inferior piezoelectric effects despite exhibiting high flexibility [152]. Therefore, there is great importance to developing composite materials that are both mechanically flexible and have a considerable piezoelectric effect. PVDF/ZnO piezoelectric sensors are a success in this direction since they allow for easy attachment to calf muscles for gait recognition and can be used to detect wrist pulses and respirations A PVDF/ZnO nanofiber-based piezoelectric sensor, enhanced by ZnO nanorods, showcases 6-fold and 41-fold sensitivity improvements in pressing and bending modes over pure PVDF, enabling precise monitoring of subtle physiological signals like respiration, wrist pulse, and muscle activity. This advancement in wearable electronics offers promising applications in health care and clinical diagnosis through its flexible, gas-permeable design and effective human physiological signal detection, highlighted by its application in a sensitive gait recognition system [153]. 

A recent study introduced an implantable PLLA/BTO piezoelectric sensor (PBPS) for real-time, long-term assessment of motor function recovery post-nerve injury, showing high biodegradability and biocompatibility. Utilizing PLLA fibers doped with BTO, the sensor converts biomechanical movements into electrical signals. Implanted in rats with sciatic nerve injury alongside tissue scaffolds, PBPS demonstrated a pressure-output voltage linearity of ≈0.9445. It accurately reflected EMG signal patterns throughout recovery, enhanced by a wireless module for unrestricted monitoring, suggesting innovative pathways for bioelectronic development in nerve repair contexts [154].

In another study, a natural composite of amino acid glycine and chitosan polymer was used to develop biodegradable piezoelectric pressure sensors for measuring pressure under wound bandages [155]. The glycine-chitosan piezoelectric films exhibit a sensitivity of approximately 2.82 mV kPa^−1^ with a capacitance range of 0.26 to 0.12 nF across 100 Hz to 1 MHz, showcasing a dielectric constant of 7.7 and a loss factor of 0.18, promising for biodegradable wearable biomedical diagnostics. The sensor demonstrated a sensitivity of 2.82 ± 0.2 mV kPa^−1^ under the pressure range of 5–60 kPa. After immersion in a pH 7.4 PBS solution for a few minutes, the sensor was completely degraded. The use of chitosan increased the flexibility of the film and controlled the crystallization of glycine into a high piezoelectric polymorph of glycine. 

### 4.4. Triboelectric Sensors

**Figure 4:**
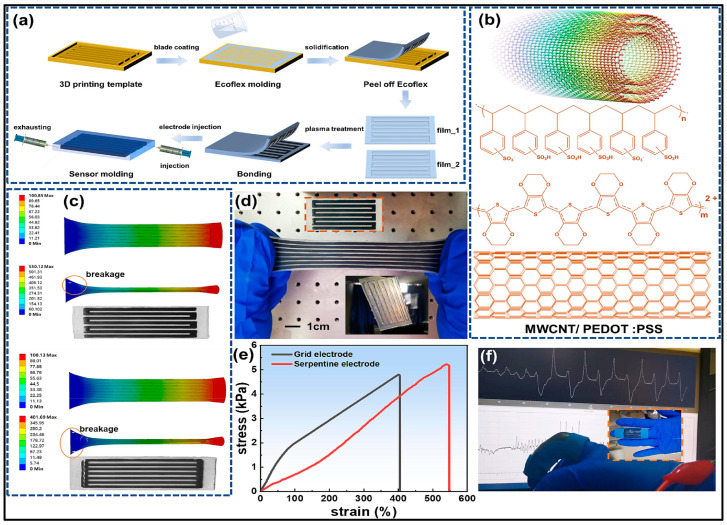
Fabrication process and structure analysis. (**a**) Fabrication process of the SLEDSS. (**b**) Schematic diagram of MWCNT-COOH structure and molecular structure of PEDOT: PSS. (**c**) Ansys finite element simulation of the serpentine and grid electrode structures against stretching strain. (**d**) Practical image of the SLEDSS in its original and stretching states. (**e**) Comparison of stress–strain curves of sensors with serpentine and grid electrode structures. (**f**) Actual finger bending test. Reprinted from *The Lancet*, ref. [157], Copyright (2024), with permission from Elsevier.

Triboelectric effects are often viewed as ubiquitous but irritating or even hazardous phenomena because of the lack of comprehensive cognition and utilization techniques. Triboelectricity occurs between any two kinds of materials, even between the same ones. It is possible to convert negligible biomechanical energy into valuable electrical energy through this universal natural event [156]. Hence, triboelectric nanogenerators (TENGs) are proposed as a power source for wearable electronic devices and as a sensor for physiological monitoring. Figure 4 details the SLEDSS fabrication, structure analysis with MWCNT-COOH and PEDOT: PSS, simulation of electrode structures under strain, stretching states images, stress-strain comparisons, and a finger bending test.

One such biodegradable triboelectric nanogenerator uses PLGA/PCL multilayered nanopatterned film and Mg electrodes to generate open-circuit voltages up to 40 V [158]. This type of device is bulky and usually loses its functionality very quickly when operated in biofluids due to its bulky design. In addition, for triboelectric harvesters, a periodic compression force would be sufficient to generate enough power for sensors to operate, but their performance is dependent on the motion or pressure of the body part to which they are attached. If, however, opposite triboelectric polarity materials are selected and the effective contact area is increased, this can amplify the triboelectric effect and can guide sensor design. The optimized FEL@CF-TENG, integrating a vulcanized silicone layer with CNTs/Ecoflex and a conductive fabric substrate, achieves impressive outputs (~490 V, ~43 μA, ~70 nC, 1.6 mW/cm^2^) under minimal force (~16 N) and frequency (~1.5 Hz), demonstrating its capability as a durable, washable, and high-performance power source for wearable electronics and electronic textiles [159]. 

Table 1 summarizes various sensors, detailing their characteristics and practical applications.

## 5. Applications of Implantable Biosensors

Many advancements in vitro diagnostics have contributed to the rise in the collection of metabolic data per patient and time, as seen by the developments in biomarkers (substances in organisms that indicate signs of conditions, diseases, or abnormal processes), and miniaturization of sensors. Such diagnostics would also result in cost savings per calculated data point being implemented by incorporating multi-parameter analytics, microfluidic technologies, and lab-on-chip systems. Although it is challenging, the extraction of data on biochemical parameters such as glucose, pH, and ionic strength from those biosensors in vivo will be helpful for diagnosis confirmation and tailoring therapy [175,176]. With personalized medicine emerging with higher demands, implantable biosensors delivering concentration transient data sets will allow for individualized care and treatment.

### 5.1. Biomarkers

#### 5.1.1. Glucose

The most crucial aspect of preventing diabetes complications is tight control of blood glucose levels. However, self-monitoring of glucose has its disadvantages. In addition to a limited number of tests that can be performed per day, it can be painful to prick fingers multiple times for sampling and the process fails to acknowledge monitoring during sleep. Continuous glucose measurement systems (CGMs) offer advantages to self-monitoring in diabetes treatment, such as the ongoing display of glucose levels. Consequently, CGMs have recently become a basic prerequisite for the individualized optimum insulin treatment of diabetics. Today, amperometric glucose biosensors, such as glucose pens and displays, are the most popular commercially available biosensors. As glucose biosensors provide near real-time self-monitoring of blood glucose levels for diabetics, their applications are numerous. Glucose biosensors make up a large part of the biosensor market and have improved the way of life for many diabetics. Additionally, biodegradable implantable glucose sensors offer less invasive, infection-reducing continuous glucose monitoring for diabetes. With no need for removal, these sensors enhance patient comfort and compliance. The natural degradation of materials ensures safety and potentially customizable lifespans, promising a sustainable and patient-friendly solution. 

CGM systems are made up of implantable electrochemical biosensors with a glucose-dependent enzyme immobilized on a microneedle, generating glucose-dependent electrical currents. Under the skin, the microneedle is inserted, and linked to a transmitter, and a separate receiver displays the glucose level. The microneedle collects blood glucose level data by measuring the glucose concentration in the subcutaneous interstitial fluid. These devices have been curated to continuously monitor changes in glucose levels for several days and then be replaced by the patient. While in use, CGM devices can send signals to diabetic patients, parents, or caregivers in the event of hypoglycemia or hyperglycemia and avoid variations beyond the usual glucose range that help avoid complications and life-threatening events [175]. Additionally, CGM devices facilitate communication between glucose sensors and insulin delivery pumps to simulate pancreatic cycles. 

In the first documented experiment involving implantable glucose biosensors, using a platinum (Pt) electrode and a dialysis membrane, glucose oxidase (GOD) was bundled against the surface of the Pt to detect oxygen by trapping it against the electrodes [177]. The activity of the enzyme varied according to the ambient oxygen concentration. The reaction of glucose with GOD creates gluconic acid, two electrons, and two protons to suppress glucose oxidase. As the reduced GOD reacts with the surrounding oxygen, electrons, and protons, hydrogen peroxide is formed, and the reaction continues to oxidize GOD back to its original form. The levels of oxygen and hydrogen peroxide are indicators that measure glucose concentrations. 

Fully implantable devices are buried under the skin using an external controller via wireless communication with interfaces. Semi-implantable systems use catheters to collect data and measurements using the micro dialysis process to draw fluid through the skin. In addition, they may also utilize other measures of fluid drawing for external glucose assay. Non-implantable devices measure glucose concentrations across the stratum corneum, and the epidermis outer layer, through spectroscopic techniques or the glucose assay through bodily fluids, such as saliva, tears, and breath. Because of their high accuracy and selectivity, implantable devices have been considered a better option. However, implantable devices come with their disadvantages. These are also associated with post-implantation inflammation, which may lead to a decreased system lifetime and involve periodic sensor adjustment by pricking the finger to draw blood for testing with another tool, defeating the intent of the biosensor’s “user-independent” design. Because of this, much research has been devoted to understanding the host tissue’s reactions to a foreign object and reducing the implant’s negative effects on the body. Research has shown that the degree of body reaction to a foreign object during implantation is proportional to the severity of the injury. Therefore, the size of the implantable device is an important consideration to reduce patient injury and discomfort and minimize inflammation of the host tissue. In a semi-implantable CGM device named Glucowizzard^TM^, the device wirelessly links to a communicator within proximity that comes with additional personal digital accessories. The device can be implanted and removed into the skin using a needle, which obviates its necessity for invasive surgery [178]. 

Minimally invasive, implantable CGM biosensors have become more prominent over the years. One such wireless hydrogel-based glucose sensor uses an inductive sensing technique for future implantable applications [179]. Under a stable DC supply, the prototype system can sense and wirelessly transmit glucose data within the human physiological range. As a protective membrane, the sensor uses electropolymerized conductive polymer polyaniline (PANI) nanofibers and a double-layer coating of polyurethane (PU) and epoxy-enhanced polyurethane (E-PU). The PU membrane regulates conveying glucose and oxygen to the sensing layer. The adhesive to the epoxy resin makes the PU membrane more durable and increases stability. 

To reduce invasiveness and risk of infection, many researchers have been developing smaller, wireless, and implantable glucose-sensing devices for glucose monitoring. One notable effort in this direction is a small, medical-grade stainless steel implantable and versatile enzyme-free CGM glucose sensor [180]. The sensor exhibits stability, low response times, good detection abilities in human plasma, and high electrocatalytic activity of glucose oxidation. A complimentary implantable glucose sensing device based on small metal-oxide-semiconductor (CMOS) image sensors deserves mention as well [133]. The device combines ultraviolet light-emitting diodes (LEDs) with optical long-path filters for measuring the fluorescence of the glucose-responsive hydrogel. The system has been used in an in vitro experiment and in acute in vivo glucose monitoring when implanted into the ear tissue of a rat. In vivo, the performance of embedded glucose sensors has been analyzed, which are filled with porous coatings treated with Dexamethasone [181]. The coating functions to moderate the tissue-sensor surface, which eventually results in reduced tissue response. Consequently, it can be inferred that this tissue microenvironment could improve the in vivo performance of glucose sensors. 

#### 5.1.2. Lactate

Lactate levels are monitored using a biosensor based on lactate oxidase and oxygen-rich platinum-doped cerium nanoparticles (Pt-ceria) during hypoxic conditions or ischemia [182]. In experiments executed in vivo on anesthetized rats, biosensors were implanted in their hippocampus and lactate concentrations were continuously monitored for 2 hrs. The Pt-ceria is a suitable substrate for bioelectrodes with implantable enzymes and measures data under hypoxic conditions. The biosensor made of these materials can be used to detect lactate rates at a very high sensitivity with a detection limit of 100 pM.

Biodegradable implantable lactate sensors are crucial for continuous lactate monitoring in critical care and are emerging as a vital tool in medical diagnostics and treatment management. These sensors, including temporary biochips offer stability and accuracy across a broad concentration range, enhancing patient care, especially in conditions like sepsis and liver disease. Their biodegradable nature reduces the need for surgical removal, mitigating long-term complications. Recent advancements in material science further reinforce the reliability of these sensors, opening new avenues for real-time disease and therapy monitoring [25,183].

A dual-responsive electrochemical transducer called, Electrochemical Cell-on-a-Chip Microdisc Electrode Array (ECC MDEA 5037), has been created and evaluated for intramuscular implantation for the continuous amperometric monitoring of lactate and glucose in an animal model [184]. Further, real-time sweat lactate dynamics have been monitored using a skin-worn lactate sensor during prolonged cycling exercises [185]. Observing temporal lactate profiles during exercise reveals insights into physical performance and general physiological health when exercising at different intensities. 

#### 5.1.3. Glutamate

Glutamate is a neurotransmitter biomarker in the brain that shapes brain development, neurotransmission, synaptic plasticity, and neurotoxicity. Glutamate is also associated with neurological disorders, such as ischemia, schizophrenia, epilepsy, Alzheimer’s disease, and Parkinson’s disease. A glutamate oxidase biosensor modified with carbon nanotubes is used to monitor glutamate flux near neurons to aid in the diagnosis of these diseases [186]. The application of such biosensors as implantable biodegradable biosensors can operate in a self-referencing (oscillating) mode during electrical stimulation to measure net glutamate flux. 

### 5.2. Central Nervous System

In situations where mild to severe Traumatic Brain Injury (TBI) occurs, invasive medical intervention becomes necessary. However, a significant concern that may arise after such intervention is brain edema [187]. This can lead to an increase in intracranial pressure (ICP), potentially impacting vital functions [188]. Various medical techniques such as sedatives, vasopressors, and antihypertensive agents, as well as surgical procedures like decompressive craniectomy and cerebrospinal fluid drainage, are employed to alleviate elevated ICP [189]. Regardless of the approach, continuous and accurate monitoring of ICP is crucial. Implantable sensors offer a solution; however, their removal requires an additional surgical procedure, which can lead to complications [190]. Biodegradable sensors present an attractive alternative as they can monitor changes in ICP within the range of 4 to 20 mmHg (normal ICP is 7–15 mmHg) [191]. In addition to pressure monitoring, these intracranial biodegradable sensors can detect temperature changes ranging from 35 to 40 degrees Celsius [132]. These temperature changes are indicative of variations in air volume inside the skull, making their tracking essential. For detecting cerebral edema associated with TBI or post-stroke vasospasm, these sensors require a minimum operational period of 14 days. To enhance their durability, biodegradable sensors have been protected using ultrathin films based on thermally grown SiO_2_, which have been shown to provide preservation for more than 22 days [128]. 

Biodegradable sensors find frequent application in the central nervous system for electrophysiological monitoring, offering a means to address various neurological disorders such as epilepsy, Alzheimer’s disease, Parkinson’s disease, depression, and chronic pain. These biodegradable neural implants serve the purpose of mapping and monitoring brain activity in different stages: before and during neurosurgery, after surgery, and following injury or drug treatment [192]. Moreover, electrophysiological sensors enable the assessment of brain damage, tracking the progress of recovery, and direct monitoring for postoperative seizures in specific surgically exposed areas of interest. 

Intracranial electrophysiological sensors measure potentials directly from the cerebral cortex using electrocorticography (ECoG). As the brain is constantly moving, the electrodes must be ultra-thin and flexible with low impedance to maintain their position and ensure high-quality signal acquisition. Si-NMs can be used as neural recording electrodes for biodegradable sensors when highly doped, as they are stable for the required short monitoring period. The electrodes are typically arranged in an array of four or six electrodes, which can be scaled to more channels. For instance, a system with 256 independent channels in a 16 by 16 configuration was built to record brain oscillations with temporal and spatial differences. While clinical studies have not been published yet, biodegradable electrical sensors implanted into rats’ intracranial space have demonstrated neuronal recordings for 33 days, with similar signal-to-noise ratios and the ability to measure brain waves and activity as standard stainless-steel sensors [127]. The electrodes are composed of Si-NMs (≈300 nm) deposited on thin PLGA substrates (≈30 μm) and encapsulated in a thin layer of SiO_2_ (≈100 nm), which provides them with the mechanical flexibility required. 

Biodegradable sensors have many other potential applications, including monitoring neurotransmitters in neural disorders such as Parkinson’s disease, attention deficit, hyperactivity, targeted drug delivery to brain tumors, and monitoring implant function and decay [193]. It has been demonstrated that electrochemical sensors made from soft biodegradable neurotransmitter molecules are useful for monitoring dopamine levels continuously and in real time. The sensors used highly doped Si NMs coated with Fe, which were electrically tuned according to the concentration of dopamine through the catalytic oxidation of the dopamine. In vitro tests using PBS immersion have shown that these sensors are sensitive in detecting dopamine concentrations as low as 10^−12^ M and selective against other neurotransmitters such as epinephrine and norepinephrine. Several sensors of this type have been developed to map dopamine secretion spatiotemporally. The combination of such neurotransmitter sensors with neural electrodes may lead to the development of new diagnostic tools for neurological diseases such as Alzheimer’s and Parkinson’s, which are characterized by chemical imbalances [194]. However, rigorous in vivo experiments are necessary to avoid injuring or interfering with the functions of sensitive brain tissue. 

### 5.3. Cardiovascular System

Following cardiovascular surgery, it is essential to confirm that blood flow through the newly created anastomosis is functioning correctly, with no leaks and minimal risk of thrombosis [146]. Currently, Doppler and skin color and turgor are the most commonly employed techniques for monitoring the patency of peripheral vessels. However, after being discharged from the hospital, patients are only monitored periodically, which may result in delayed detection of vascular patency issues, leading to tissue, graft, or patient loss. Additionally, the use of Doppler systems with ultrasonic probes is limited to hospitals because they require wired connections, which require a secondary surgery to remove [195]. It is therefore possible to monitor vessel patency continuously using biodegradable blood flow sensors implanted during surgery. 

To solve the problem, a biodegradable pressure sensor patch has been created. The patch utilizes a microstructured dielectric layer made of poly(glycerol sebacate) (PGS) between two magnesium electrodes to form a capacitive structure [196]. This device has been effectively employed to measure blood pulse waves in human arteries, with the skin improving the signal-to-noise ratio (SNR) and response time. In addition, the measurement of arterial blood flow in a healing vessel was achieved with a self-powered biodegradable sensor using fringe-field double capacitor structures [195]. 

### 5.4. Musculoskeletal System

**Figure 5:**
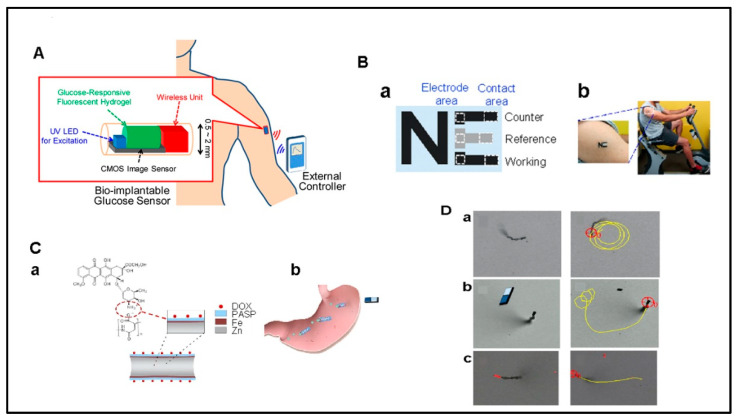
(**A**) A CMOS-based implantable glucose-sensing system using glucose-responsive fluorescent hydrogel [198] © 2024 Optical Society of America. (**B**) (**a**) Schematic illustration of a three-electrode “NE” tattoo biosensor for electrochemical epidermal monitoring of lactate. (**b**) An “NE” lactate biosensor applied to a male volunteer’s deltoid. Adapted with permission from ref. [185], Copyright 2013 American Chemical Society. (**C**) Schematic structure of a microrocket (**a**) and its application for effective localization in the stomach (**b**) Adapted with permission from ref. [199], Copyright 2019, American Chemical Society. DOX = Doxorubicin, PASP = Poly (aspartic acid). (**D**) (**a**) Self-propulsion of PASP/Fe–Zn microrocket; (**b**) magnetic navigation of PASP/Fe–Zn micro rocket; (**c**) self-propulsion of DOX/PASP/Fe–Zn micro rocket suspended in the gastric acid simulant (pH = 1.2) with surfactant [151]. The yellow trajectories indicate the motion. Scale bar 30 μm, adapted with permission from ref. [199], Copyright 2019 American Chemical Society.

Musculoskeletal injuries often require surgical treatment and careful monitoring to repair both hard (bones) and soft tissues (tendons, skin, muscles) and restore their function. To ensure positive outcomes and reduce patient distress during recovery, it is important to measure the physiological pressures exerted on tendons and muscles after surgery and implement personalized recovery strategies accordingly. However, current musculoskeletal pressure monitoring methods, such as MRI and ultrasound, are not suitable for continuous real-time monitoring [142]. Although devices for continuous monitoring are available, they are currently limited in their clinical usefulness because they were designed for biomechanical laboratory use [197]. Figure 5 showcases a CMOS-based glucose sensor, a lactate-monitoring tattoo biosensor, microrocket structures for stomach localization, and their propulsion and navigation mechanisms in acidic environments.

Biodegradable sensors for measuring strain and pressure on soft tissue are typically piezoelectric or capacitive. Self-powered piezoelectric sensors rely on the mechanical deformation of piezoelectric PLLA to generate a signal through the current that it produces. Capacitive sensors, on the other hand, measure strain by monitoring changes in capacitance caused by the movement of two thin film comb electrodes of Mg sliding past each other [142]. 

### 5.5. Force Sensing

The accurate measurement of force within biological systems is crucial for a wide range of applications, from monitoring cardiac activity to measuring the pressure exerted by growing tissues. Two prominent sensing mechanisms utilized for this purpose are piezoelectricity and piezoresistivity, each offering unique advantages.

Piezoelectric materials generate an electrical charge in response to mechanical stress, making them highly suitable for force sensing applications. Their high sensitivity and rapid response time are beneficial for dynamic measurements. Materials like polyvinylidene fluoride (PVDF) and zinc oxide (ZnO) are commonly used piezoelectric materials in bioelectronic devices due to their biocompatibility and efficient piezoelectric properties [200,201]. Piezoresistive sensors alter their electrical resistance when subjected to mechanical strain. Silicon nanowires (SiNWs) and conductive polymers are frequently employed as piezoresistive materials. These sensors are known for their precision in static force measurements and compatibility with microfabrication techniques, making them ideal for integrating into wearable and implantable devices [202,203]. Both sensing mechanisms have been effectively applied in the design of bioelectronic devices to measure forces that are relatively small, yet critical for understanding physiological conditions. By leveraging the piezoelectric or piezoresistive properties of materials, bioelectronic devices can provide valuable insights into biomechanical processes, offering opportunities for advanced diagnostic and therapeutic applications [204,205].

### 5.6. Other Applications

The use of biodegradable sensors is not limited to monitoring vital parameters for diagnosis, but they are also useful for delivering drugs to tumors [206]. Since the blood–brain barrier prevents the penetration of drugs into brain tumors, externally controlled biodegradable implants loaded with drugs offer an attractive tool for targeted drug delivery to the brain. To achieve controlled intracranial drug delivery via thermal stimulation, a biodegradable device consisting of a polymer-drug reservoir, a heater, and a temperature sensor has been developed and tested both in vitro and in dogs’ brains. The use of hydrophilic/hydrophobic bifacial design enabled the flexible biodegradable device to adhere conformally to the brain tissue, decrease tumor volume, and improve survival rate. The device disintegrated in 10 weeks without leaving any residue or causing any side effects [206]. 

Kim and coauthors proposed a noninvasive method to detect uric acid in saliva samples using a wearable electrochemical mouthguard biosensor [207]. For selective and robust saliva analysis, screen-printed electrodes were modified with a uricase enzyme and protected with a polymeric layer. The authors demonstrated that the proposed device could monitor uric acid concentrations in healthy and hyperuricemic individuals. Using this clever approach, it is possible to monitor the evolution of salivary uric acid during appropriate drug treatment over four consecutive days. In the gastrointestinal tract, biodegradable origami-based robots that can be ingested into the stomach have been deployed that locomote to a desired location, remove a foreign body, patch a wound, deliver drugs, and eventually biodegrade [208,209]. Microrockets are similar microdevices that are composed of poly (aspartic acid) microtubes, thin Fe intermediate layers, and Zn cores. They can carry drugs, magnetically locate targets, penetrate gastric mucus gels, increase the retention of drugs in the stomach without causing obvious toxic reactions, and eventually become decomposed by gastric acid or proteases in the digestive tract [199,210].

## 6. Challenges and Future Directions

### 6.1. Power Supply

Implantable sensors, crucial in medical diagnostics and treatment, face challenges in power supply, essential for their functionality. Balancing energy consumption with device size is key, as these sensors need compact, efficient power sources. Solutions include energy harvesting from the body and external sources, along with energy storage and wireless power transfer, considering power constraints and device dimensions [211]. Radio Frequency Energy Harvesting (RFEH) is emerging as a promising power source for wearable and implantable medical devices due to its non-invasive nature and ambient energy utilization, though efficiency and device integration challenges persist. Similar advances in smart wearables offer potential applications for implantable sensors, providing insights that could be transferable to implantable devices [212,213].

Moreover, integrating sensors into networks necessitates energy harvesting and wireless power transfer for sustainable power. Techniques like harnessing magnetic fields near power lines and thermoelectric generators (TEGs) are emerging. These innovative methods, converting body heat to electricity, show promise in adequately powering sensors and enhancing the performance and lifespan of implantable medical devices [214,215,216].

The development of biodegradable sensors for clinical applications is constrained by low energy consumption and appropriate device size. To provide a fully functional electrical system in implantable biodegradable devices, bioresorbable power supplies are required, such as batteries, energy harvesters, or flexible circuits. Biodegradable batteries using biodegradable Mg and Mo/Fe as electrodes, electrode space filled by PBS or NaCl solution, and encapsulated by polyanhydride/PLC polymer have been reported [11]. These batteries could output an average power of 30 Watts for 100 h and operate at 1.6 V. However, battery life was limited to a few hours to a few days following immersion in biofluid solutions at 37 °C. A notable advancement in energy storage devices is the use of sweat or sweat-equivalent solutions as electrolytes [217]. 

Another option for powering biodegradable sensors is to use energy harvester devices, particularly for devices that require low power consumption and long-term operation. In this regard, piezoelectric and triboelectric generators, which generate energy from the mechanical movement of the body, are ideal for in vivo energy harvesting. In one study, piezoelectric zinc oxide strips were deposited on silk substrates, and Mg electrodes were used as electrodes for a biodegradable piezoelectric harvester [218].

Piezoelectric transducers can be utilized to take advantage of daily bodily motions to produce energy. There are two types of human body motions: discontinuous and continuous. Discontinuous motion includes walking or hand movements, actions that are not always occurring. Continuous motion includes breathing or blood flow, things that our bodies do naturally. Implementation of piezoelectric transducers into moving body parts has been investigated, from joints, and muscle switches to shoes. Research has found that continuous motions result in lower power levels when compared to discontinuous motions which require more range of motion. 

Because significant movements are needed to generate enough power with piezoelectric transducers, a select few body parts are good candidates for implantation in vivo (knee, foot, or elbow). Piezoelectric generators have been proposed for in vivo applications. For instance, PZT-5A and polyvinylidene fluoride plates were proposed to generate power from blood pressure fluctuations [219], and piezoelectric ceramics were proposed to be implemented in knee replacement implants [220]. However, piezoelectric transducers have also been applied outside of the body. For example, Kymissis et al. piezoelectric transducers have been integrated into shoe heels and were able to deliver 1 W of power [221]. 

Biodegradable triboelectric nanogenerators can generate voltages of up to 40 volts using nanopatterned PLGA and PCL films and Mg electrodes [158]. Zhong Lin Wang et al. utilized implantable triboelectric nanogenerators (iTENG) in vivo to monitor the heart wirelessly [222]. Another version of the item by the same authors designed a device that converts the mechanical energy from a rat’s breathing into electricity. That energy harvested was then stored in a capacitor and used to operate a pacemaker to control the heart rate of a rat. This work makes it clear that they can turn biomechanical energy into electricity. Another group proposed a tribe-NG backpack, capable of harvesting energy by walking with the load. The backpack is made up of polytetra-flurorethylene and aluminum plates with a PET substrate [223]. When the layers come in contact, power is generated. This has also been integrated into a shoe insole. 

Recent reports have also highlighted sweat-based biofuel cells as a promising advanced energy generation solution [176,224]. All the above energy devices have the drawback of being bulky when compared to the sensor itself, losing their functionality quickly when placed in biofluids, and not being flexible enough. Furthermore, piezoelectric/triboelectric harvesters would also provide sufficient power under periodic compression for sensors to function, but their performance is largely dependent on the motion or pressure of the implanted part. 

### 6.2. Data Communication

Implantable sensors, essential in personalized medicine and early disease detection, encounter significant challenges in data communication. Advancements in miniaturization, biocompatibility, sensor capabilities, and wireless communication are crucial for these devices. However, ensuring data security and privacy in wireless body area networks (WBANs) is an important issue, with a focus on secure and dependable data storage and fine-grained access control [225,226]. High-speed data communication development is also pivotal for new medical devices [227]. 

In vivo, data communication differs from laboratory phantoms and tissue models, and it presents unique challenges for implantable sensors [228]. This includes the need to overcome areas of penalty-degraded antenna matching and poor signal-to-noise ratio [229]. Additionally, ensuring secure communication in wireless biosensor networks is vital, given their limitations in power, memory, and computation [230]. Wireless power transfer and communication using WiTricity technology for biomedical sensors and implants are being explored to improve efficiency and transmission distance. Trust-based models in Wireless Body Area Networks (WBAN) have been proposed to ensure secure communication within networks. These developments highlight the complexities and the need for ongoing research to enhance data communication in implantable sensors [231,232].

In biodegradable sensors, data are communicated simply by thin wires connected to a external circuitry and power supply [146]. For biomedical applications, the wires can increase infection risks and limit mobility. Several biodegradable sensors have been developed with fully transient wireless technologies to improve mobility and reduce infection risk [196]. The devices are generally wireless data transmitters that use resonant inductive coupling between the external circuitry and the implanted device to transmit data [233]. These passive inductive systems have a simple structure, low weight, and prevent tissue damage from power dissipation by enabling battery-free operation. However, these devices are limited to short-distance communication (a few millimeters), and the measured signal is highly dependent on the intermediate tissue and coil position [234]. Additionally, the sensed signals are mainly limited to a shift in the resonant frequency of the external coil, and the operation is limited to a short frequency range. As a result of these issues, these sensors are limited in their potential applications, for instance in situations where implants must be placed deep within the brain or inside the body. 

### 6.3. Materials

Implantable sensors encounter significant challenges regarding materials, impacting their functionality and compatibility with human tissues. Hydrogel-based sensors for bioelectronics, offering electron conductivity, antibacterial properties, and tissue adhesion, are able to address these challenges. Developing pressure sensors that are safe for long-term biological tissue interfaces poses another challenge. Flexible and biodegradable materials for chemical sensors also present difficulties in materials and device design. Spintronic-based magnetoresistive sensing technology offers potential solutions for detecting magnetomyogram signals in miniaturized wearable and implantable systems. These challenges underscore the need for innovative materials to enhance the performance and integration of implantable sensors [235,236,237,238].

The biodegradable sensors have limited available options for material selection and correlate with intended application, in vivo degradation rates, and compatibility with existing fabrication techniques. New materials that are to be used for semiconductors, conductors, dielectrics, and encapsulation are in the scope of future biodegradable implantable sensors. Since existing materials are rigid, researchers need to develop biodegradable semiconducting materials that are flexible and have high conductivity. Notably, metals have poor conductivity, or conductive polymers are difficult to synthesize. In addition, to maintain the stable operation of a biodegradable sensor for clinically relevant times, it is crucial to encapsulate the sensor to protect it from body fluids. Another challenge is choosing the right encapsulating material, since biodegradable polymers such as PLGA or silk fibroin are prone to swelling, resulting in premature capsule fractures and water ingress [239]. Fabricating encapsulations without micro defects with alternative materials such as SiO_2_ or metal oxides is difficult [240]. As a result, new materials and fabrication techniques, such as the thermal growth of SiO_2_, can improve sensor encapsulation properties and durability.

### 6.4. Fabrication

Fabricating implantable sensors presents several challenges, including the need for biocompatibility, flexibility, and thin materials, especially for applications in small regions. Advances in nanomaterials and printing technologies are important, yet they pose specific hurdles in implantable applications. Additionally, the mismatch between rigid circuit materials and soft human tissues necessitates the use of polymeric and biodegradable metallic materials. These challenges highlight the complexity and innovation required in the fabrication process of implantable sensors [241,242,243].

The fabrication of biodegradable sensors is challenging since they must conform to soft and irregular tissue contours. Furthermore, conventional microfabrication techniques cannot be applied to most biodegradable materials due to their dissolvability at and sensitivity to high temperatures. To manufacture biodegradable sensors, new fabrication techniques have been developed, such as soft lithography, screen printing, and transfer printing [244,245]. Lithography-based techniques, however, cannot produce fine sensor microstructures and complex interconnections. On the other hand, printing-based methods are expensive and take a long time to fabricate. 

Despite the limited number of techniques available for biodegradable sensor fabrication, many studies have successfully demonstrated the manufacturing of sensor components [132,133,246] and a few studies have also been successful in demonstrating the application of these devices in vivo [132,142]. Recently, CMOS technologies have been used to produce electronics on Si-on-Insulator wafers with wafer-scale and foundry compatibility [239]. This method considerably reduces expenses, although it involves time-consuming vacuum processes [127]. Improvements in 3D- and 4D-printing techniques will allow for low-cost, scalable, reliable, and reproducible fabrication. This will pave the way for biodegradable sensor commercialization. 

### 6.5. Implanting into Body

Implanting sensors poses challenges, including optimizing inductive antenna design for wireless use ensuring device size reduction without compromising sensitivity, and addressing biocompatibility, power supply, and regulatory considerations in total hip replacements [247,248,249].

Biocompatible, flexible, and thin materials are required for implantation in small regions. Post-implantation functionality loss due to foreign body response and the danger of infection from artificial components like wires are significant concerns [241,250,251]. These challenges underscore the complexity of implanting sensors effectively and safely. 

In the last 50 years, implantable biodegradable electronics have made significant progress in terms of reliability, thanks to advances in encapsulation and packaging that protects indwelling modules from hostile environments [252]. Nonetheless, due to the invasive nature of many surgical procedures and the weakened immune response frequently observed among implant recipients, implant-associated infections and inflammation are highly prevalent in these individuals [253]. Typically, biomaterial-associated infections develop from peri- and post-operative contaminations, which are the most common routes of introduction of etiological agents as well as via the bloodstream [254].

**Figure 6:**
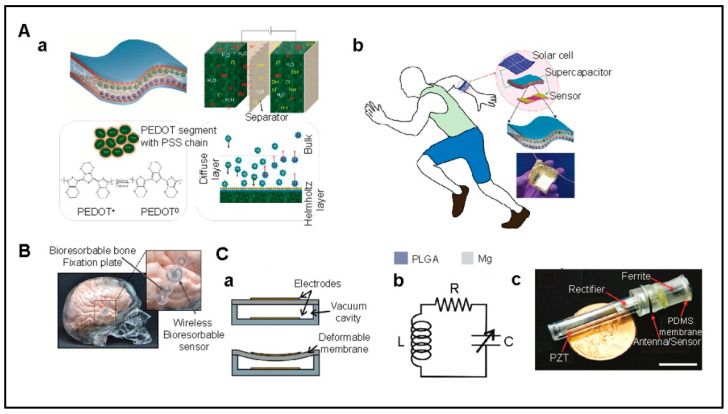
(**A**) Power Supply: Schematic representation and mechanism of the PEDOT: PSS coated on cellulose cloth, which absorbs sweat as an electrolyte for wearable supercapacitors (**a**) and its application in humans. (**b**) Figure reproduced with permission from ref. [217], copyright 2020, WILEY-VCH Verlag GmbH & Co. KGaA, Weinheim. (**B**) Data communication: bioresorbable passive resonance sensors based on inductor–capacitor (LC) circuits providing wireless readout. Figure reproduced with permission from ref. [234], Copyright 2013, American Chemical Society. (**C**) Long-term performance and calibration: schematic of operation of a capacitive-based membrane pressure sensor (**a**), circuit model of passive LC tank (**b**), and inductor-based sensor with piezoelectric energy harvester for measurements of bladder pressure. (**c**) Adapted with permission from ref. [259] under the terms of the Creative Commons Attribution CC by 4.0 Copyright MDPI Sensors 2014.

Depending on the severity, complications may range from pain requiring localized antibiotic treatment to removing the device and administering systemic antibiotics [255]. Among the reasons for the relatively high rate of implant-associated infections is the fact that the implant surface is non-living, which makes it an ideal colonization environment for bacteria and its inability to send chemical signal warnings to the surrounding tissues to stay alert. The initial stages of bacterial attachment can be mitigated by certain combinations of surface properties; however, bacterial cells have been shown to release extracellular polymeric substances to precondition surfaces that otherwise would not be suitable for habitation, rendering these measures ineffective. Consequently, antibacterial drug-releasing surfaces that prevent bacterial adhesion and replication and destroy attached bacteria are receiving considerable attention. In addition to traditional antibiotics, an array of alternative coatings and antimicrobials have been studied, including silver ions, nitric oxide, bioactive antibodies, and other bactericidal compounds [256], resulting in a significant reduction in patient cost and morbidity. Figure 6 explores wearable supercapacitors powered by sweat-absorbing PEDOT: PSS on cellulose, bioresorbable wireless sensors, and long-term performance of bladder pressure monitoring systems with energy harvesting.

Since most implantable biodegradable devices include polymers, metals, and composites, it should be considered how different types of degradation fragments contribute to degradation. It was found that both polymer and metallic debris activated macrophages and giant cells in the peri-implant area, resulting in tissue loss [257]. Because of their differences in size, metallic particles are more mobile and more easily transferred from the peri-implant space to other tissues and organs, thus activating immune cells and eventually triggering an inflammatory response. In contrast, large, irregularly shaped ultra-high-molecular-weight polymer particles are less mobile, with an inclination to accumulate in tissues close to the implant site. Using encapsulation, implant surfaces have been made biocompatible and integrated with host tissues more effectively, resulting in precise control of degradation kinetics and cytocompatibility of degradation byproducts, which result in fewer infections at surgical sites [126]. However, it is crucial to understand that the behavior of biodegradable material is primarily affected by its environment (e.g., pH, ion concentrations, oxygen) [258]. Hence, it is imperative to investigate the cytocompatibility, biocompatibility, and potential toxicity of the degradation by-products of biodegradable electronic devices to make these exciting technologies available for clinical use. 

### 6.6. Long-Term Performance and Calibration

Implantable sensors face significant challenges in long-term performance and calibration. These include optimizing calibration models for accuracy and bias reduction and addressing sensitivity issues due to device size reduction [248,260]. Calibration approaches, such as next-generation implantable glucose monitoring systems, demonstrate sustained accuracy and safety. These complexities highlight the need for ongoing innovation in implantable sensor technology [261].

Biodegradable sensors implanted in humans should have stable, consistent responses over their lifetime to perform reliable long-term measurements. In sensors, signal drift can occur in two ways: (1) offset drift, in which the base measurement slowly drifts to obscure the desired measurement, and (2) sensitivity drift, where the sensitivity of the device slowly decreases with time [259]. The causes of drift can be attributed to changes in the environment such as tissue encapsulation and changes independent of the environment such as material aging and mechanical fatigue. This is why implanted sensors should have drift compensation circuits or zeroing functions to provide reliable measurements over time. 

Comparing the sensor signal with a controlled reference is a usual method to remove drift in the baseline measurement. A capacitor implanted alongside the sensing element that is unresponsive to changes in the desired parameter can be employed as a reference in a differential signal circuit that subtracts common effects of drift [262]. A variation of this concept is to average the output from several sensors in an array to compensate for drift, which may give advantages such as enhancing the range and sensitivity of the system. 

Calibration of sensor responses in the in vivo environment may be facilitated by adding temperature data. Increasing the long-term performance of these sensors is possible using onboard temperature compensation circuitry [263] or external signal processing circuitry [264]. In comparison to non-degradable sensors, calibration is often harder for biodegradable sensors and the functional lifetime always varies a little bit with every individual case. It can be hard to confirm when the accurate functional lifetime is over [196]. 

### 6.7. Integrated Design Considerations

The design and development of these implantable biodegradable biosensors entail a multifaceted approach that encompasses power supply, data communication, and implantation strategies, closely intertwined with the selection of materials and fabrication methods. This section aims to present an integrated overview of these elements, highlighting the recent progress while acknowledging the persistent challenges. The efficacy of implantable sensors is fundamentally tied to their power supply mechanisms, which must be compact yet efficient due to the inherent limitations in device size and the need for minimal energy consumption. Innovations such as Radio Frequency Energy Harvesting (RFEH) and energy harvesting from body movements—via piezoelectric and triboelectric generators—represent significant strides in sustainable power sourcing. These technologies not only harness ambient and biological energy but also promise compatibility with biodegradable components, such as Mg and Mo/Fe electrodes for batteries and nanopatterned PLGA and PCL films for triboelectric nanogenerators [25,265].

The degradation rate of materials is pivotal in determining the sensor’s lifespan within the body. Materials like PLGA (Poly(lactic-co-glycolic acid)) and polyanhydride are selected for their predictable biodegradation rates, which can be tailored to match the desired lifespan of the sensor. Biodegradable batteries using Mg and Mo/Fe as electrodes, encapsulated by polyanhydride/PLC polymer, exemplify the strategic selection of materials that degrade at a rate compatible with the device’s operational lifespan, ensuring that the device functions effectively until its intended end of life [266]. Simultaneously, ensuring reliable data communication in a body-implanted sensor demands advancements in miniaturization and wireless technology, underpinned by secure, efficient data transmission protocols. Biodegradable sensors leverage thin wires or wireless data transmitters based on resonant inductive coupling, emphasizing the need for materials and designs that support short-distance, high-fidelity data communication without compromising biocompatibility or increasing infection risks [267]. Materials selection for these sensors is critical, with a focus on biodegradability, compatibility with human tissues, and functionality. The use of hydrogel-based sensors, flexible and biodegradable conductors, and encapsulation materials like polyanhydride and PLC polymers underscores the importance of material innovation in overcoming challenges related to sensor integration, longevity, and performance within the body. These materials provide electron conductivity, antibacterial properties, and tissue adhesion, making them suitable for long-term applications in biological environments [268]. Fabrication techniques for implantable biodegradable sensors must address the intricacies of working with materials that are sensitive to conventional processing methods. Techniques such as soft lithography, screen printing, and transfer printing have emerged as viable approaches to produce sensors capable of conforming to soft tissue contours, albeit with challenges in achieving microstructural precision and cost-effectiveness. The implantation of biodegradable sensors into the body remains a complex procedure that necessitates careful consideration of device size, sensitivity, and biocompatibility [25,269,270].

The integration of real-time monitoring systems with pacemakers represents a significant leap forward in cardiac care, enabling continuous and remote patient health tracking. A notable advancement is the BlueSync technology by Medtronic, which facilitates the direct monitoring of pacemakers using smartphones or tablets through an app-based platform, showing a higher rate of transmission success compared to traditional methods. This technology empowers patients with direct access to their pacemaker data and has been demonstrated to potentially reshape patient monitoring practices [271]. Similarly, groundbreaking work by researchers at Northwestern University introduced a smart, dissolving pacemaker that is part of a body-area network of sensors for monitoring physiological functions. This network not only allows for the autonomous detection of abnormal cardiac rhythms but also enables the pacemaker to communicate with patients through haptic feedback, enhancing the care experience by promoting recovery in the comfort of the patient’s home [272]. These innovations underscore the potential of integrated monitoring systems in providing sophisticated, patient-centric cardiac care [273,274,275,276].

In summary, the design and development of implantable biodegradable biosensors necessitate a holistic approach where power supply, data communication, and implantation strategies are intricately linked with the choice of materials and fabrication methods. This integrated framework ensures that the resulting devices are not only tailored to the functional requirements of in-body sensing but also aligned with the principles of biodegradability and tissue compatibility.

### 6.8. Future Directions

As we gaze into the horizon of implantable biodegradable sensors, we stand on the cusp of a transformative era in medical diagnosis and therapeutic monitoring. The future directions of these cutting-edge devices are poised to be shaped by several key trends and innovations.

#### 6.8.1. Material Science Advances

The development of new biodegradable materials with enhanced properties, such as improved biocompatibility, increased mechanical strength, and controlled degradation rates, is likely to expand the applicability of biosensors. Research is moving towards the synthesis of novel polymers and composites, including smart materials that respond to specific physiological triggers to degrade only when required. Furthermore, the integration of these advanced materials into biosensor platforms promises to bridge critical gaps in current healthcare diagnostics by enabling real-time, in situ monitoring of physiological states with unprecedented accuracy and sensitivity. Research efforts are also geared towards harnessing the potential of nanotechnology to fine-tune the surface properties of biosensors, enhancing their interaction with biological systems for improved performance. The exploration of bioresorbable electronics that seamlessly integrate into the body’s environment without eliciting adverse immune responses represents a vital area of development. Ultimately, these material science innovations will redefine the boundaries of implantable biodegradable biosensors, offering more personalized, predictive, and preventive healthcare solutions.

#### 6.8.2. Nanotechnology Integration

Nanotechnology promises to revolutionize implantable biosensors by enabling the creation of nanostructured materials that provide high surface area, superior sensitivity, and multifunctionality, crucial for detecting minute physiological changes with high precision. Nanosensors could provide real-time monitoring at the cellular or even molecular level, offering unprecedented insights into physiological processes and the early detection of diseases. Moreover, the application of nanotechnology in biosensors facilitates the integration of multiple diagnostic functions within a single platform, enabling simultaneous monitoring of various biomarkers. This multi-analyte detection capability is vital for comprehensive health assessments and personalized medicine. Additionally, nanoengineering surfaces can significantly improve the biocompatibility and longevity of implantable devices, reducing the risk of immune rejection and enhancing patient safety. As research progresses, the convergence of nanotechnology with other emerging fields, such as bioinformatics and synthetic biology, is expected to further expand the diagnostic and therapeutic potential of implantable biosensors, pushing the frontiers of what is currently achievable in medical technology.

#### 6.8.3. Wireless and Energy Harvesting Technologies

The incorporation of wireless communication and energy harvesting capabilities will facilitate the development of biosensors that can transmit data in real time while being powered by body-derived energy sources, such as heat or motion. This will result in sensors that are minimally invasive and self-sustaining. The capability to convert physiological activities into usable energy not only ensures continuous operation but also opens avenues for long-term health monitoring without the need for battery replacements or surgical interventions. Furthermore, the adoption of wireless communication technologies in biosensors facilitates seamless data transmission to external devices, enabling real-time health monitoring and rapid diagnostic feedback. This wireless connectivity supports the integration of biosensors into telehealth systems, promoting remote patient monitoring and personalized healthcare management. The development of energy-efficient wireless protocols and the optimization of energy harvesting mechanisms are critical to maximizing the longevity and functionality of these devices. As research advances, the fusion of these technologies promises to yield biosensors with unparalleled operational independence, ushering in a new era of medical diagnostics that are both minimally invasive and highly informative.

#### 6.8.4. Personalized Medicine

Personalized medicine will benefit from biosensors tailored to individual patient’s genetic profile and medical histories. Such biosensors will offer the potential to monitor physiological parameters and detect disease markers with unprecedented precision, thereby enabling highly personalized healthcare interventions. By accounting for genetic variations that influence disease susceptibility and treatment response, these sensors will help in the early detection of pathologies, optimize therapeutic strategies, and minimize adverse drug reactions. Moreover, the integration of biosensors in personalized medicine will facilitate continuous health monitoring, providing real-time insights into a patient’s health status and the effectiveness of prescribed treatments. This dynamic approach to healthcare will not only enhance disease management but also empower patients by involving them more closely in their health decisions. As we move towards more individualized healthcare paradigms, the role of advanced biosensors becomes increasingly important. Future research and development in this area are likely to focus on improving sensor specificity, reducing costs, and ensuring seamless integration with digital health platforms to fully realize the promise of personalized medicine.

## 7. Conclusions

Self-degradability of biosensors and their capabilities to monitor in situ biochemical/biomechanical conditioning are essential to embrace the modern approach of data-driven diagnosis. The development of implantable biodegradable sensors hinges on biocompatibility, miniaturization, and reliability. Designing biodegradable devices and ensuring uniform mechanical/electrical behavior is a challenging endeavor. Biosensors need to be in synergy with the host environment, evade the immune response, and degrade harmlessly within a clinically approved duration through natural biological processes. Secondly, the fabrication methodology needs to incorporate sensing units, power supply, communication systems, and encapsulation that is fully biodegradable. In addition, clinical acceptance and material stability are of major concern. The choice of metals is interconnected to the functionality, operating period, and physiologically permitted concentration. On the other hand, polymers have problems with molecular weight disintegration and unwanted fluid penetration. Fabrication poses a challenge to provide intricate morphology and flexibility to match the surrounding tissue contour. Post-implant infections, inflammation, and contamination are prone to instigate implant surfaces becoming a suitable habitat for bacteria. Sensor performance decreases due to material aging, mechanical weariness, and tissue encapsulation. The calibration of implantable biodegradable sensors is a complex task as determining functional lifetime depends on multifarious factors. This review emphasized the alternative emerging biodegradable materials that have a high impact on further aiding the development of implantable sensors. Substrates made from plant cellulose have eminent potential though their functionality and adaptability in an aqueous environment require additional research. Silk as a substrate ticks all the boxes for applications in drug delivery to food sensing technology, both inorganic and organic semiconductors have their pros and cons for active layers. Materials discussed in this article have application-specific characteristics. Therefore, sensor design should accentuate the purpose, location, and environment to achieve real-world applications.

## Figures and Tables

**Table 1:** Different sensors, their attributes, and applications.

Sensor Type	Analyte	Physiological Range	Detection Limit	Application	In Vivo/In Vitro	Highlight
Resistive Sensor [137]	Acetone	Under 0.6 mmol/L (177 ppb to 2441 ppb)	0.1 to 2 ppm	Detecting acetone directly in the exhaled breath	Exhaled breath	Detects acetone directly in the exhaled breath.
Resistive Sensor [138]	Ethanol	Less than 50 mg/dL, or 0.05% concentration	5 ppm–1000 ppm	Gas sensing	Homes and industrial sites	Alcohol sensor with high selectivity and stability.
Resistive Sensor [160]	Pressure	Flexible	10–500 kPa	Sensing subtle pressure levels like pulse pressure and high-pressure levels like fingertip pressure	Conformal contact with the skin	Ultrathin, biocompatible, and flexible pressure sensor.
Resistive Sensor [161]	Ammonia	29–688 ppb, Avg 265 ppb	0.2 to 10 ppm	Detecting ammonia directly in the exhaled breath	Exhaled breath	Molecularly modified. SnO_2_ sensors for sensing polar gases such as ammonia.
Resistive Sensor [162]	Avidin	0.05% of total protein (1800 μg/egg)	0.1 nM (6.8 ng⁄ml)	Detection of protein (Avidin)	Lab based	Detection of protein in low concentrations.
Capacitive Sensor [163]	Static pressure	Flexible	Compression 0.09/N, Sheer 0.06/(a.u), Bending 0.06/(a.u)	Detection of static pressure	Measures static pressure (cannot be done by piezoelectric sensors)	Highly flexible pressure sensors.
Capacitive Sensor [164]	Gram-force	Stretchable	GF = 0.7 Pressure sensitivity 1.62 MPa^−1^	Simultaneously detects stretch, pressure, temperature, or touch	Finger and knee	Highly sensitive and wearable sensors.
Capacitive Sensor [165]	Gram-force	Stretchable	GF = 1	Strain gauges to detect human motion	Onto smart clothes or directly onto the body	Date glove, monitoring of balloon inflation and chest movement.
Capacitive Sensor [166]	Pressure	Stretchable	0.7 kPa^−1^ (0–1 kPa), 0.14 kPa^−1^ (1–5 kPa), 0.005 kPa^−1^ (5–20 kPa)	Pressure sensing	Electronic Skin	The first stretchable energy-harvesting electronic skin device.
Capacitive Sensor [167]	Pressure	Flexible	5.54 kPa^−1^ (0–30 Pa), 0.88 kPa^−1^ (30–70 Pa)	Monitoring of knee/finger bending, forearm muscular movement and air blow	Finger, arms, and knee	The sensor has been readily integrated into an adhesive bandage and has been successful in detecting human movements.
Piezoelectric Sensor [168]	Pressure	Flexible	0.1 Pa–2 KPa	Pressure sensing	With the human body or with advanced robotic systems	The ability to bend and stretch is attractive for pressure/force sensors.
Piezoelectric Sensor [146]	Pressure	Flexible	0–18 kPa	Pressure sensing	Monitoring of pressure in various parts of the body such as the brain, lungs, eyes, and heart	A biodegradable implantable pressure sensor using PLLA.
Piezoelectric Sensor [155]	Pressure	Flexible	5–60 kPa	Pressure sensing	Measuring pressure under wound bandages	Amino acid glycine and chitosan polymer-based biodegradable pressure sensors.
Piezoelectric Sensor [169]	Pressure	Flexible	0.23 to 10 kPa	Pressure sensing	Human skin	Force-sensing resistors and field effect transistor (FET) sensors for monitoring biological pressure and force-sensing.
Piezoelectric Sensor [170]	Piezoelectric Coefficient	Flexible	4.7–6.4 pC/N	Piezoelectric response	Ambient condition	Potential applications in the fields of electronics, sensors, and biomedical diagnostics.
Triboelectric Sensor [158]	Ammonia	29 to 688 ppb	50–10,000 ppm	Detection of ammonia in breath	Breath analysis	Monitoring of exhaled gases in human breath for disease diagnosis.
Triboelectric Sensor [171]	Acetone, Toluene	0.3–1.0 ppm Acetone, 6.5 ± 1.5 ppm Toluene	35 ppb, 3.0 ppm for acetone 1 ppb,10 ppm for toluene	Detection of acetone and toluene in breath	Breath analysis	Volatilome analyzer consisting of polymer nanofiber-MWCNT composite that responds to acetone and toluene.
Triboelectric Sensor [172]	Alcohol	0.01–0.5% (40–500 mg/dL)	10–2000 ppm	Detection of alcohol in breath.	Breath analysis	Breathed-out alcohol concentration detection regardless of the blow speed and quality of airflow.
Triboelectric Sensor [173]	Lactate	6.5 mM (forehead) to 13 mM (foot) with an avg 5.9 mM for the whole body	10 µM–20 mM	Detection of lactate in sweat	Sweat analysis	Lightweight and fully self-powered electrochemical sensing system to manage and self-monitor lactate concentration in sweat.
Triboelectric Sensor [174]	Glucose	0.001–5.50 mM	0.1–1 mM	Onto the clothes	Lab testing	Nonenzymatic glucose detection.
